# Integrated Functional Characterization of a Panel of Clinical Orthoflavivirus Isolates Reveals Distinct Replication and Innate Immune Response Profiles in Human Keratinocytes

**DOI:** 10.3390/v18080826

**Published:** 2026-07-27

**Authors:** Tannya Karen Castro Jiménez, Edwin Antonio Lopez Kelly, Leticia Cedillo-Barrón, Julio García-Cordero, Diego Sait Cruz-Hernández, Nallely Diaz Lima, José Alberto San Juan Luis, Cruz Carlos Castillo Camacho, Eloy Andrés Pérez-Yépez, Cynthia Daniela Ibarra-Moreno, Luis Angel Flores-Mejía, Sergio Roberto Aguilar-Ruíz, Mónica G. Mendoza-Rodríguez, Luis I. Terrazas, José Bustos-Arriaga

**Affiliations:** 1 Laboratorio de Biología Molecular e Inmunología de arbovirus, Unidad de Biomedicina, Facultad de Estudios Superiores Iztacala, Universidad Nacional Autónoma de México, Tlalnepantla 54090, Mexico; tannya.castro@iztacala.unam.mx (T.K.C.J.); edwinkelly99@gmail.com (E.A.L.K.); 2 Departamento de Biomedicina Molecular, Centro de Investigación y de Estudios Avanzados del Instituto Politécnico Nacional, Zacatenco 07360, Mexico; lcedillo@cinvestav.mx (L.C.-B.);; 3 Departamento de Ciencias Biomédicas, Universidad de la Sierra Sur, Guillermo Rojas Mijangos s/n, Esq. AV. Universidad, Col. Ciudad Universitaria, Miahuatlán de Porfirio Díaz, Oaxaca 70800, Mexico; cruzdiegos@hotmail.com; 4 OaxacaLab Laboratorio de Análisis Clínicos, Oaxaca 68000, Mexico; 5 Laboratorio Ecología Microbiana, UBIPRO, Facultad de Estudios Superiores Iztacala, Universidad Nacional Autónoma de México, Tlalnepantla 54090, Mexico; biocruzccc@iztacala.unam.mx; 6 Laboratorio de Señalización Celular e Inmunología del Cáncer (SECIC), Instituto Nacional de Cancerología, San Fernando 22, Col. Sección XVI, Tlalpan, Ciudad de México 14080, Mexico; eperezy2306@gmail.com (E.A.P.-Y.); a00846033@tec.mx (C.D.I.-M.); 7 Escuela de Medicina y Ciencias de la Salud, Tecnológico de Monterrey, Ciudad de México 14380, Mexico; 8 Laboratorio de Falla Medular y Carcinogénesis, Departamento de Medicina Genómica y Toxicología Ambiental, Sede Periférica del Instituto de Investigaciones Biomédicas, Universidad Nacional Autónoma de México, Coyoacán, Ciudad de México 04530, Mexico; luisangel.flores_mejia@iibiomedicas.unam.mx; 9 Torre de Investigación del Instituto Nacional de Pediatría, Av. del Imán No1, Insurgentes Cuicuilco, Coyoacán, Ciudad de México 04530, Mexico; 10 Departamento de Biomedicina Experimental, Facultad de Medicina y Cirugía de la Universidad Autónoma ‘Benito Juárez’ de Oaxaca, Oaxaca 68120, Mexico; saguilar.cat@uabjo.mx; 11 Unidad de Biomedicina, Facultad de Estudios Superiores Iztacala, Universidad Nacional Autónoma de México, Avenida de los Barrios 1, Los Reyes Iztacala, Tlalnepantla 54090, Mexico; monica.mendoza@iztacala.unam.mx (M.G.M.-R.); literrazas@unam.mx (L.I.T.)

**Keywords:** Orthoflavivirus, dengue virus, Zika virus, West Nile virus, clinical isolates, keratinocytes

## Abstract

Orthoflaviviruses comprise genetically diverse mosquito-borne viruses responsible for a broad spectrum of human diseases. Although naturally circulating clinical isolates exhibit biological variability, the extent to which they generate distinct early epithelial innate immune responses remains incompletely understood. Here, we characterized five clinical orthoflavivirus isolates obtained in Oaxaca, Mexico, using human HaCaT keratinocytes as an in vitro model of early infection. Productive infection was assessed by immunofluorescence microscopy, immunostained focus appearance under isolate-optimized assay conditions, and infectious virus production, whereas host responses were evaluated by transcriptional profiling and quantitative whole-slide single-cell immunofluorescence. All isolates established productive infection and exhibited different viral replication profiles. Temporal transcriptional analyses revealed variable expression of antiviral (IFNβ, Mx1, OAS1, PKR, IFITM3, Viperin, and RANTES) and inflammatory (TNF-α, IL-8, and MCP-1) genes. Quantitative whole-slide analysis provided complementary evidence of variable STAT1 and NF-κB signaling activation across the analyzed isolates. Within this limited panel, viral replication was not consistently aligned with the selected transcriptional and signaling readouts, although the exploratory nature of these comparisons precludes establishing independence between these variables. Together, the virological, transcriptional, and imaging analyses revealed distinct multidimensional functional profiles across the isolate panel. Overall, these findings demonstrate functional heterogeneity among the analyzed clinical orthoflavivirus isolates and highlight integrated functional phenotyping as a useful framework for examining virus–host interactions beyond viral replication alone.

## 1. Introduction

Orthoflaviviruses are arthropod-borne, positive-sense, single-stranded RNA viruses that include several pathogens of major public health importance, including dengue virus, Zika virus, West Nile virus, yellow fever virus, Japanese encephalitis virus, and tick-borne encephalitis virus. Most members of the genus Orthoflavivirus are transmitted by mosquitoes or ticks and replicate in both arthropod vectors and vertebrate hosts, facilitating their maintenance in complex ecological cycles and their emergence in human populations [1].

Orthoflavivirus infection is initiated in the skin, where keratinocytes, fibroblasts, endothelial cells, macrophages, dendritic cells, and other resident immune cells contribute to the earliest antiviral and inflammatory responses. Keratinocytes are not merely structural cells; they support viral replication and produce type I and type III interferons, chemokines, and inflammatory mediators following infection with viruses such as dengue virus, West Nile virus, and Zika virus. These early epithelial responses are likely to influence local viral amplification, immune-cell recruitment, systemic dissemination, and ultimately disease outcome [2,3].

Orthoflaviviruses have evolved multiple strategies to evade innate immune responses. A major target of viral immune antagonism is the interferon signaling axis, particularly the JAK/STAT pathway. Several mosquito-borne orthoflaviviruses interfere with type I interferon signaling through viral non-structural proteins, including NS5, although the underlying mechanisms vary among viral species and strains. This immune antagonism reduces STAT1 activation, impairs interferon-stimulated gene expression, and promotes viral replication despite the presence of antiviral cues [4,5].

In parallel, NF-κB signaling plays a central role in the induction of inflammatory cytokines and chemokines during orthoflavivirus infection. Although this response contributes to antiviral defense and immune-cell recruitment, excessive or dysregulated NF-κB activation may also promote tissue inflammation and immunopathology. Consequently, the balance between antiviral interferon signaling and inflammatory NF-κB activation represents a key determinant of host–virus interactions, viral fitness, and pathogenic potential [6,7].

Although the biology of prototype laboratory strains has been extensively investigated, considerably less is known about the functional diversity of contemporary clinical orthoflavivirus isolates. Clinical isolates circulating in endemic regions may differ in replication capacity, phenotypic characteristics, and their ability to modulate host innate immune signaling pathways. Such phenotypic variability may be particularly relevant in regions where multiple orthoflaviviruses co-circulate and where ecological, vector, and host factors facilitate repeated spillover and transmission [8,9].

In this study, we characterized five clinical orthoflavivirus isolates obtained in Oaxaca, Mexico, using human HaCaT keratinocytes as an epithelial model of early infection. We integrated viral replication kinetics, immunofluorescence detection of infected cells, transcriptional profiling of antiviral and inflammatory genes, and quantitative analysis of STAT1 and NF-κB p65 phosphorylation. This integrative approach allowed us to determine whether differences among clinical isolates are limited to replicative fitness or whether they also involve distinct patterns of innate immune response profiles.

Our findings demonstrate that clinical orthoflavivirus isolates establish productive infection in keratinocytes but differ in immunostained focus appearance, replication kinetics, antiviral gene induction, inflammatory gene expression, and activation of STAT1 and NF-κB signaling. Because the isolate panel included representatives of three orthoflavivirus species, this study was designed to compare their functional behavior rather than to distinguish isolate-specific effects from species-specific biological characteristics. Collectively, these findings indicate that the phenotypic diversity observed among the analyzed isolates exhibited distinct host-response profiles that were not consistently aligned with viral replication efficiency but also reflects differential temporal modulation of innate immune pathways.

## 2. Materials and Methods

### 2.1. Clinical Isolates and Molecular Confirmation

Five clinical orthoflavivirus isolates obtained in Oaxaca, Mexico, were included in this study: Zika virus (ZIKV) isolates 2019.Oax.10 and 2019.Oax.19; West Nile virus (WNV) isolates 2019.Oax.17 and 2016.Oax.02; and dengue virus serotype 2 (DENV-2) isolate 2019.Oax.31. The viruses were isolated from serum samples collected from patients presenting with acute febrile illness who had initially been screened using rapid immunochromatographic assays for the DENV NS1 antigen and for DENV- and ZIKV-specific IgM and IgG antibodies, as previously reported [8]. The clinical isolates were obtained from serum samples collected from febrile patients in Oaxaca, Mexico, between September 6, 2016, and December 2021, as part of an ongoing research project on arboviral infections. The study involving human participants was initially reviewed and approved by the Research and Ethics Committee of the Master’s Program in Experimental Biomedicine, Faculty of Medicine, Universidad Autónoma “Benito Juárez” de Oaxaca (UABJO), where patient recruitment and sample collection were conducted. Subsequent processing and characterization of the isolates at the Facultad de Estudios Superiores Iztacala, Universidad Nacional Autónoma de México (UNAM), were conducted with the approval of the Ethics Committee of the Facultad de Estudios Superiores Iztacala (approval code CE/FESI/112017/1224; approved on 17 November 2017).

Written informed consent was obtained from all participants or, when applicable, from their legal guardians before sample collection. All samples were anonymized before laboratory analysis, and the investigators had no access to personally identifiable information. All procedures were conducted in accordance with the ethical principles of the Declaration of Helsinki.

The isolates were selected from our institutional collection to represent the orthoflavivirus species identified in Oaxaca during the study period. Selection criteria included successful virus isolation, confirmation of viral identity by RT-PCR, sufficient availability of viral stocks, and reproducible infectivity in cell culture. Isolates were not selected according to clinical severity or patient outcome. Their main characteristics are summarized in Table 1.

Before experimentation, all isolates were propagated under standardized laboratory conditions in Vero cells to generate working stocks. All infection experiments were conducted using molecularly confirmed, low-passage viral stocks at passage 3 (P3), thereby minimizing potential phenotypic changes associated with extensive in vitro propagation (Table 1). Viral species and serotype identities were confirmed by RT-PCR using commercially available multiplex assays based on hydrolysis probes: the Logix Smart™ ZDC assay (Co-Diagnostics, Salt Lake City, UT, USA) and the Liferiver Dengue Virus Genotyping Real-Time RT-PCR Kit (Shanghai ZJ Bio-Tech Co., Shanghai, China). WNV identity was confirmed using a previously published probe-based RT-PCR protocol [10].

### 2.2. Cell Culture

Human immortalized keratinocytes (HaCaT) were maintained in high-glucose Dulbecco’s Modified Eagle Medium (DMEM; HyClone Cytiva, Logan, UT, USA) supplemented with 10% heat-inactivated fetal bovine serum (FBS; HyClone Cytiva, Logan, UT, USA) and 1% penicillin–streptomycin (HyClone Cytiva, Logan, UT, USA). Vero cells were maintained in Roswell Park Memorial Institute medium (RPMI 1640; HyClone Cytiva, Logan, UT, USA) supplemented with 5% FBS and 1% antibiotic–antimycotic solution. Cells were incubated at 37 °C in a humidified atmosphere containing 5% CO_2_. Cell cultures were routinely passaged at 70–80% confluence and periodically confirmed to be free of mycoplasma contamination.

### 2.3. Viral Propagation and Titration

Viral stocks were propagated in Vero cells. Culture supernatants were harvested when a cytopathic effect was evident and clarified by centrifugation at 3000× *g* for 10 min to remove cellular debris. Viral stocks were aliquoted and stored at −80 °C until use. Each aliquot was thawed only once before infection experiments. Infectious viral titers were determined by focus-forming unit (FFU) assay in Vero cells.

### 2.4. Infection of Keratinocytes

HaCaT cells were seeded to achieve approximately 80% confluence at the time of infection. Cells were infected with each clinical isolate at a multiplicity of infection (MOI) of 1. Viral inocula were prepared in DMEM supplemented with 5% FBS and added to the cell monolayers for 1 h at 37 °C with gentle rocking every 15 min to ensure uniform virus distribution. Following adsorption, the inoculum was removed and replaced with fresh DMEM containing 5% FBS.

Samples were collected at 6, 12, 24, and 48 h post-infection (hpi). For each isolate and time point, three independent biological replicates were performed on separate experimental days.

### 2.5. Focus Formation Units Assay

Viral titers in viral stocks and supernatants collected from infected HaCaT cultures were determined by a focus-forming unit (FFU) assay in Vero cells, as previously described [8]. Briefly, confluent Vero cell monolayers seeded in 24-well plates were infected with 10-fold serial dilutions of each viral sample. After 1 h of adsorption at 37 °C, the inoculum was removed, and the cells were overlaid with 1% methylcellulose (Sigma-Aldrich, St. Louis, MO, USA) prepared in RPMI 1640 supplemented with 5% FBS.

Plaque assay incubation times were optimized according to the growth characteristics of each viral isolate to ensure reliable focus enumeration. Isolates 2019.Oax.10 and 2019.Oax.19 were incubated for 5 days because plaques remained too small for reliable enumeration after 3 days, whereas the remaining isolates were fixed and processed after 3 days. All viral isolates were processed using the same immunostaining protocol, including the primary anti-flavivirus envelope monoclonal antibody 4G2 and the corresponding secondary antibody, to ensure consistent detection conditions throughout the study [8].

### 2.6. RNA Extraction and RT-qPCR

Total RNA was extracted from infected and mock-infected HaCaT cells using the RNeasy Mini Kit (QIAGEN, Hilden, Germany), according to the manufacturer’s instructions. Complementary DNA (cDNA) was synthesized using the RevertAid M-MuLV Reverse Transcriptase Kit (Thermo Fisher Scientific, Waltham, MA, USA), following the manufacturer’s recommendations. Quantitative PCR was performed using REALQ Plus 2× Master Mix Green (Ampliqon A/S, Odense, Denmark) on a MIC Real-Time PCR System (Bio Molecular Systems, Upper Coomera, QLD, Australia).

Gene-specific primer pairs for IFNB1, MX1, OAS1, EIF2AK2 (PKR), IFITM3, and RSAD2 (Viperin) were obtained from commercially validated qPCR primer sets (OriGene Technologies, Rockville, MD, USA). These primer sets were amplified using the cycling conditions recommended by the manufacturer: an initial enzyme activation step at 95 °C for 10 min, followed by 40 cycles of 95 °C for 15 s and 60 °C for 1 min. A melting-curve analysis was performed at the end of each run to verify amplification specificity. Primer pairs for TNF, IL8, CCL2 (MCP-1), CCL5 (RANTES), and GAPDH were obtained from previously published studies and amplified using the cycling conditions reported in the corresponding publications [11,12,13,14]. Primer sequences, amplification conditions, sources, and corresponding references or catalog numbers are provided in Supplementary Table S1.

All reactions were performed in technical triplicate, and no-template controls were included in each run. Relative gene expression was calculated using the comparative 2^−ΔΔCt^ method, with GAPDH as the endogenous reference gene. Each infected sample was normalized to its corresponding time-matched non-infected control collected at the same post-infection time point (6, 12, 24, or 48 hpi), which served as the calibrator for relative quantification. Independent positive-control cultures were included to verify the responsiveness of the RT-qPCR assays. For antiviral gene induction, cells were treated with recombinant human IFN-α A/D (10 or 100 IU/mL; Sigma-Aldrich, St. Louis, MO, USA) for 6, 12, 24, or 48 h. Two IFN-α A/D concentrations were included to evaluate antiviral gene responsiveness across different levels of type I interferon stimulation. For inflammatory gene induction, separate cultures were treated with lipopolysaccharide from Escherichia coli O111:B4 (LPS; 1 µg/mL; Sigma-Aldrich, St. Louis, MO, USA) for 6 h. These treatment groups were analyzed independently from the viral infection experiments and are presented in Supplementary Table S2. Because they served as assay validation controls rather than experimental comparison groups, they were not included in the integrated heatmap analysis. GAPDH was selected as the endogenous reference gene based on its widespread use in RT-qPCR studies of flavivirus infection, and its Ct values were examined during data analysis to verify the absence of substantial variation across the experimental conditions evaluated. Because the objective of this study was to compare relative transcript abundance among experimental groups, absolute quantification and standard curves were not performed.

### 2.7. Immunofluorescence Staining

For immunofluorescence analyses, 5 × 10^4^ HaCaT cells were seeded onto sterile glass coverslips placed in 24-well plates. After 24 h, the cells were infected with each clinical orthoflavivirus isolate at an MOI of 1 under the same conditions used for the transcriptional analyses. Infected cultures were fixed at 12 h post-infection to evaluate early signaling events corresponding to the transcriptional analyses. Mock-infected cells were included as negative controls.

Recombinant human IFN-α A/D (100 IU/mL; Sigma-Aldrich, St. Louis, MO, USA) for 20 min was used as a positive control for STAT1 activation because type I interferons induce canonical JAK–STAT signaling. Lipopolysaccharide from Escherichia coli O111:B4 (LPS; 1 µg/mL; Sigma-Aldrich, St. Louis, MO, USA; kindly provided by Dr. Araceli Pérez-López, UNAM, México) for 2 h was used as a positive control for NF-κB activation because it is a well-established inducer of inflammatory signaling. Positive controls were included in every independent staining experiment and processed in parallel with infected samples under identical staining conditions.

At the indicated time points, cells were washed twice with phosphate-buffered saline (PBS) and fixed with freshly prepared 4% paraformaldehyde (Sigma-Aldrich, St. Louis, MO, USA) for 15 min at room temperature. Samples were permeabilized with 0.1% Triton X-100 (Sigma-Aldrich, St. Louis, MO, USA) in PBS for 10 min and blocked with 5% bovine serum albumin (BSA; Sigma-Aldrich, St. Louis, MO, USA) in PBS for 1 h to reduce nonspecific antibody binding.

Primary antibodies against phosphorylated STAT1 at Tyr701 and phosphorylated NF-κB p65 at Ser536 (Cell Signaling Technology, Danvers, MA, USA) were incubated overnight at 4 °C. After three washes with PBS, samples were incubated with the appropriate fluorophore-conjugated secondary antibodies (Invitrogen, Thermo Fisher Scientific, Waltham, MA, USA) for 1 h at room temperature in the dark. Nuclei were counterstained with DAPI (Invitrogen, Thermo Fisher Scientific, Waltham, MA, USA), and the actin cytoskeleton was stained with phalloidin (Thermo Fisher Scientific, Waltham, MA, USA). Coverslips were mounted using VECTASHIELD antifade mounting medium (Vector Laboratories, Inc., Newark, CA, USA).

All staining procedures were performed simultaneously for the experimental groups included in each staining set, using identical fixation, permeabilization, antibody-incubation, washing, and mounting conditions.

### 2.8. Image Acquisition

Fluorescence images were acquired using two complementary imaging platforms. Whole-slide fluorescence images were obtained using a Zeiss Axio Scan.Z1 digital slide scanner (Carl Zeiss AG, Oberkochen, Germany), whereas representative high-magnification micrographs were acquired using a Zeiss Axio Scope (Carl Zeiss AG, Oberkochen, Germany) fluorescence microscope equipped with 40× and 100× objectives.

Images were acquired sequentially using the DAPI, Alexa Fluor 488, and Alexa Fluor 547 channels. Exposure time, illumination intensity, detector gain, and camera settings were maintained constant for all samples within each staining experiment. Images were exported in OME-TIFF format without nonlinear contrast enhancement before quantitative analysis.

Representative images shown in the figures were selected exclusively for visualization and were not used for fluorescence quantification.

### 2.9. Whole-Slide Image Processing and Quantitative Fluorescence Analysis

Whole-slide fluorescence images were processed using a tissue-based cyclic immunofluorescence image-analysis workflow adapted from previously described pipelines. Raw fluorescence images were first corrected for illumination heterogeneity using the BaSiC algorithm. Corrected image tiles were subsequently stitched and spatially registered using ASHLAR, generating registered whole-slide OME-TIFF images for quantitative analysis.

Single-cell segmentation was performed independently for each of the three biological replicates using the DAPI nuclear channel and the StarDist deep-learning algorithm. The resulting nuclear masks were used to identify individual cells throughout each complete scanned specimen. Fluorescence intensity was then quantified at the single-cell level across all acquired channels. Mean fluorescence intensity (MFI) was extracted for each segmented cell within every whole-slide image and exported as comma-separated value files for downstream analysis.

For each biological replicate, the median MFI across all segmented cells was calculated to generate one representative value per replicate. These replicate-level median MFI values, obtained from three independent biological replicates per condition, were used as the experimental units for all statistical comparisons.

Violin plots were generated by pooling the single-cell MFI values from the three independent biological replicates to visualize the distribution and heterogeneity of fluorescence intensity within each experimental condition. These pooled single-cell data were used exclusively for visualization and were not used for statistical inference.

Image preprocessing, stitching, registration, segmentation, and fluorescence quantification followed the BMF t-CyCIF Image Processing Pipeline for multiplex immunofluorescence image analysis. Representative whole-slide fluorescence images acquired using the Zeiss Axio Scan.Z1 are shown in Supplementary Figure S2. Quantitative fluorescence measurements were obtained from complete scanned specimens rather than from manually selected microscopic fields.

### 2.10. Statistical Analysis and Data Integration

All experiments were performed using three independent biological replicates unless otherwise stated. RT-qPCR reactions were performed in technical triplicate, and the mean Ct value for each biological replicate was used for downstream analysis. Viral titers were log10-transformed before statistical comparison. Relative gene-expression values were calculated using the 2^−ΔΔCt^ method and log2-transformed for visualization.

Replication kinetics and transcriptional data were analyzed using two-way ANOVA, with isolate and time post-infection as independent variables, followed by Tukey’s multiple-comparisons test when appropriate. Complete results of the RT-qPCR statistical analyses, including pairwise comparisons, adjusted *p*-values, and significance levels, are provided in Supplementary Table S3.

For quantitative immunofluorescence analyses, the median MFI obtained from each independent biological replicate was used as the experimental unit (*n* = 3 biological replicates per condition). Statistical comparisons were performed using the Kruskal–Wallis test followed by Dunn’s multiple-comparisons test. Single-cell fluorescence-intensity values were pooled exclusively for visualization in violin plots and were not used for statistical inference.

Associations between viral replication and host gene expression were evaluated using Spearman’s rank correlation coefficient. For integrated phenotypic analyses, selected variables—including viral titers at 24 and 48 hpi, antiviral and inflammatory gene expression, and signaling readouts—were standardized using Z-score normalization according to the following formula:

Z = (x − μ)/σ

where x represents the value obtained for a given isolate, μ represents the mean across isolates, and σ represents the corresponding standard deviation.

Heatmaps were generated in GraphPad Prism using mean log2-transformed values or Z-scores and displayed using a diverging red–white–blue color scale centered at zero. No clustering algorithm was applied. Instead, isolates were manually ordered according to their phenotypic profiles to facilitate descriptive biological interpretation. Replicate-level median fluorescence intensity values and the corresponding statistical comparisons are provided in Supplementary Figure S3 and Tables S4 and S5.

All statistical analyses were performed using GraphPad Prism version 11. Statistical significance was defined as *p* < 0.05 unless otherwise indicated.

## 3. Results

### 3.1. Clinical Orthoflavivirus Isolates Establish Productive Infection and Exhibit Distinct Infection Phenotypes

**Figure 1:**
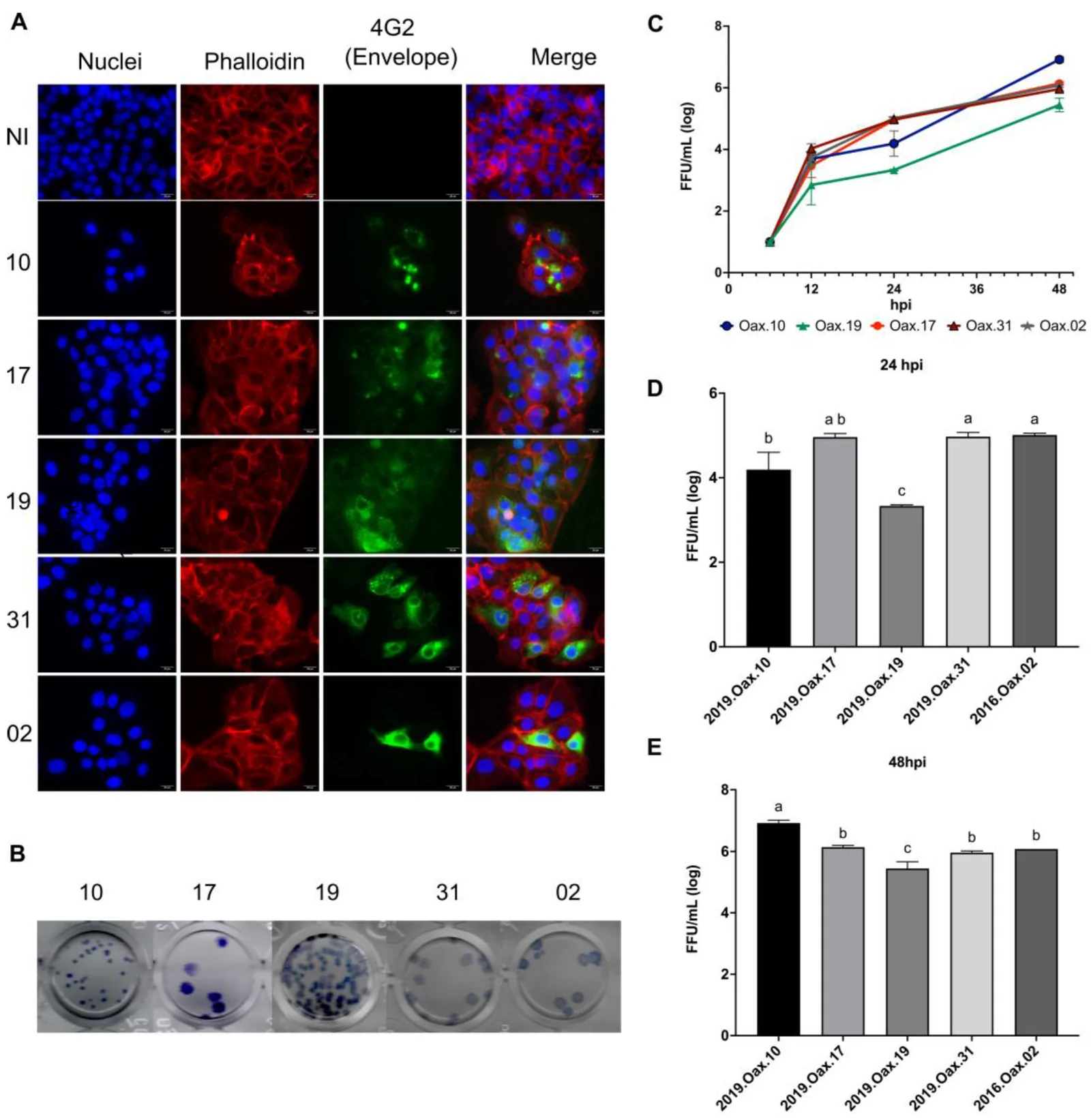
Clinical orthoflavivirus isolates establish productive infection and exhibit distinct infection phenotypes in human keratinocytes. (**A**) Representative immunofluorescence micrographs of HaCaT cells infected with the five clinical orthoflavivirus isolates at a multiplicity of infection (MOI) of 1 for 48 hpi. Nuclei were stained with DAPI (blue), F-actin with phalloidin (red), and viral antigen was detected using the pan-flavivirus monoclonal antibody 4G2 (green). Merged images illustrate productive infection and differences in the distribution of infected cells among isolates. Scale bar = 20 µm. (**B**) Representative plaque assay wells illustrating the characteristic immunostained focus appearance of each clinical isolate. Plaque assays were performed using identical immunostaining conditions; isolates 2019.Oax.10 and 2019.Oax.19 were fixed after 5 days because plaques remained too small for reliable enumeration at 3 days, whereas the remaining isolates were processed after 3 days. (**C**) Replication kinetics of the five clinical orthoflavivirus isolates in HaCaT cells. Infectious virus production was quantified by focus-forming assay at 6, 12, 24, and 48 h post-infection (hpi) and expressed as log10 focus-forming units (FFU)/mL. Data represent the mean ± SD of three independent biological replicates. (**D**,**E**) Viral titers at 24 hpi (**D**) and 48 hpi (**E**) presented as individual biological replicates. Different lowercase letters denote statistically significant differences among isolates as determined by two-way ANOVA followed by Tukey’s multiple-comparison test (*p* < 0.05). The analyzed clinical isolates are summarized in Table 1.

To determine whether the selected clinical orthoflavivirus isolates established productive infection in human epithelial cells, HaCaT monolayers were infected at a multiplicity of infection (MOI) of 1 and evaluated by immunofluorescence microscopy at 48 hpi, together with quantification of infectious virus production by the focus-forming unit (FFU) assay at 6, 12, 24, and 48 hpi (Figure 1).

Immunofluorescence analysis confirmed productive infection for all five clinical orthoflavivirus isolates, as demonstrated by the detection of intracellular viral antigen in infected cultures (Figure 1A). Representative micrographs revealed differences in the distribution of infected cells, intracellular fluorescence intensity, and the organization of infection foci, indicating heterogeneous infection phenotypes despite identical experimental conditions.

Phenotypic variability was further supported by differences in the appearance of immunostained foci observed during viral titration (Figure 1B). Although all isolates produced readily detectable foci under identical immunostaining conditions, variations in focus appearance were observed under isolate-optimized assay conditions. Because incubation time was extended only when necessary to obtain reliable focus enumeration, these observations should be interpreted as qualitative characteristics of each isolate under its optimized assay conditions rather than as evidence of differential viral spread or spreading phenotypes.

To characterize viral replication, infectious virus production was quantified at 6, 12, 24, and 48 hpi (Figure 1C). All isolates produced infectious progeny throughout the experimental period, confirming that HaCaT cells supported productive replication. Viral titers increased progressively over time for all isolates; however, the magnitude of viral amplification differed among isolates as infection progressed.

Statistical comparisons of viral titers revealed significant differences among the analyzed isolates at both 24 and 48 hpi (Figure 1D,E). At 24 hpi, isolate 2019.Oax.19 produced significantly lower viral titers than isolates 2019.Oax.31 and 2016.Oax.02, whereas the remaining isolates exhibited intermediate replication levels. By 48 hpi, isolate 2019.Oax.10 reached the highest viral titers, whereas 2019.Oax.19 remained significantly lower than all other isolates. Despite these differences, several isolates displayed partially overlapping replication profiles, indicating that all isolates established productive infection but differed in the extent of viral amplification over the course of infection.

Overall, these findings demonstrate that all clinical orthoflavivirus isolates successfully established productive infection in human keratinocytes while exhibiting differences in infection phenotype, immunostained focus appearance, and viral replication kinetics. These observations provide the foundation for subsequent analyses aimed at determining whether differences in viral replication are accompanied by distinct antiviral and inflammatory host responses.

These representative images are presented to illustrate immunostained focus appearance under isolate-optimized assay conditions and should not be interpreted as evidence of differential viral spread or spreading phenotypes because incubation times differed among isolates.

### 3.2. Clinical Orthoflavivirus Isolates Exhibit Distinct Temporal Antiviral Responses and STAT1 Activation

**Figure 2:**
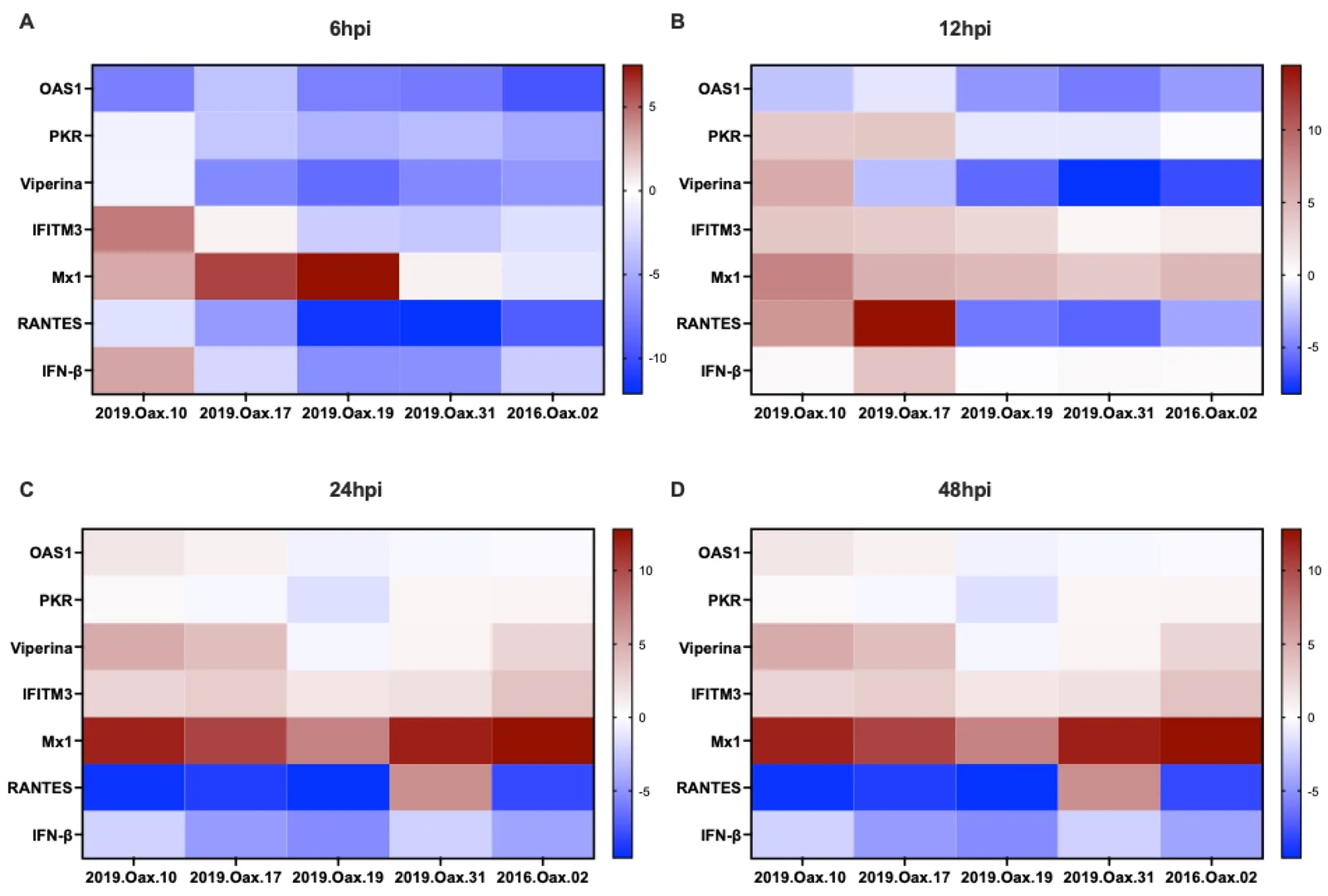
Clinical orthoflavivirus isolates differentially regulate antiviral gene expression in human keratinocytes. Relative expression of IFN-β, Mx1, OAS1, PKR, IFITM3, Viperin, and RANTES was determined by relative RT-qPCR in HaCaT cells infected with the five clinical orthoflavivirus isolates (MOI = 1). Gene expression was evaluated at 6 hpi (**A**), 12 hpi (**B**), 24 hpi (**C**), and 48 hpi (**D**). Expression values were calculated using the comparative 2^−ΔΔCt^ method, with GAPDH as the endogenous reference gene. Relative expression values at each post-infection time point were normalized to the corresponding time-matched non-infected (NI) control processed in parallel, which served as the calibrator. Data are presented as log_2_ (2^−ΔΔCt^) values from three independent biological experiments. The individual biological replicate values are provided in Supplementary Table S2, and the complete statistical analyses are available in Supplementary Table S3.

To characterize the antiviral response elicited by the five clinical orthoflavivirus isolates, the transcriptional expression of genes associated with the type I interferon (IFN-I) pathway was evaluated at 6, 12, 24, and 48 h post-infection (hpi). The antiviral transcriptional panel included IFN-β, Mx1, OAS1, PKR, IFITM3, Viperin, and RANTES (Figure 2).

Distinct temporal transcriptional profiles were observed throughout the course of infection. At the earliest time points (6 and 12 hpi), IFN-β expression differed in both timing and magnitude among isolates, indicating heterogeneous activation of the primary interferon response (Figure 2A,B). Some isolates exhibited early IFN-βinduction, whereas others showed delayed or comparatively weaker expression.

A similar pattern was observed for interferon-stimulated genes (ISGs). Expression of Mx1, OAS1, PKR, IFITM3, and Viperin increased progressively throughout infection; however, both the magnitude and kinetics of induction varied among isolates (Figure 2). Although transcript levels remained relatively low at 6 hpi, differences became more apparent at 12 hpi and were further accentuated at 24 and 48 hpi. Among the ISGs evaluated, Mx1 displayed the most consistent induction at later time points, whereas Viperin, OAS1, PKR, and IFITM3 exhibited greater variability in both temporal dynamics and expression levels.

The interferon-inducible chemokine RANTES also displayed differences in temporal expression across the analyzed isolates. Some isolates induced sustained expression throughout infection, whereas others maintained comparatively lower transcript levels, further illustrating differences in activation of interferon-associated antiviral programs.

To determine whether these transcriptional responses were accompanied by activation of the canonical interferon-signaling pathway, STAT1 phosphorylation was evaluated by quantitative immunofluorescence (Figure 5). Representative fluorescence micrographs revealed differences in phosphorylated STAT1 (p-STAT1) staining among infected cultures (Figure 5A). Quantitative analysis, based on automated whole-slide image segmentation and fluorescence-intensity measurements, showed that all clinical isolates induced detectable STAT1 phosphorylation relative to basal controls, although the magnitude of activation differed significantly among isolates (Figure 5C). IFN-α A/D-treated cells exhibited the expected increase in p-STAT1 fluorescence intensity and served as positive controls for pathway activation.

Overall, the combined transcriptional and quantitative immunofluorescence analyses indicate that the analyzed clinical orthoflavivirus isolates exhibit distinct antiviral response profiles in human keratinocytes. The STAT1 phosphorylation analyses provide an independent functional readout of interferon signaling that complements the longitudinal transcriptional data obtained at multiple post-infection time points. Because phosphorylation was evaluated only at 12 h post-infection, these measurements should be interpreted as an early signaling snapshot rather than as temporal validation of the transcriptional responses. The individual biological replicate RT-qPCR values underlying these analyses are provided in Supplementary Table S2. Complete statistical results, including the two-way ANOVA followed by Tukey’s multiple-comparisons test, are presented in Supplementary Table S3.

### 3.3. Clinical Orthoflavivirus Isolates Differentially Regulate Inflammatory Responses and NF-κB Activation

**Figure 3:**
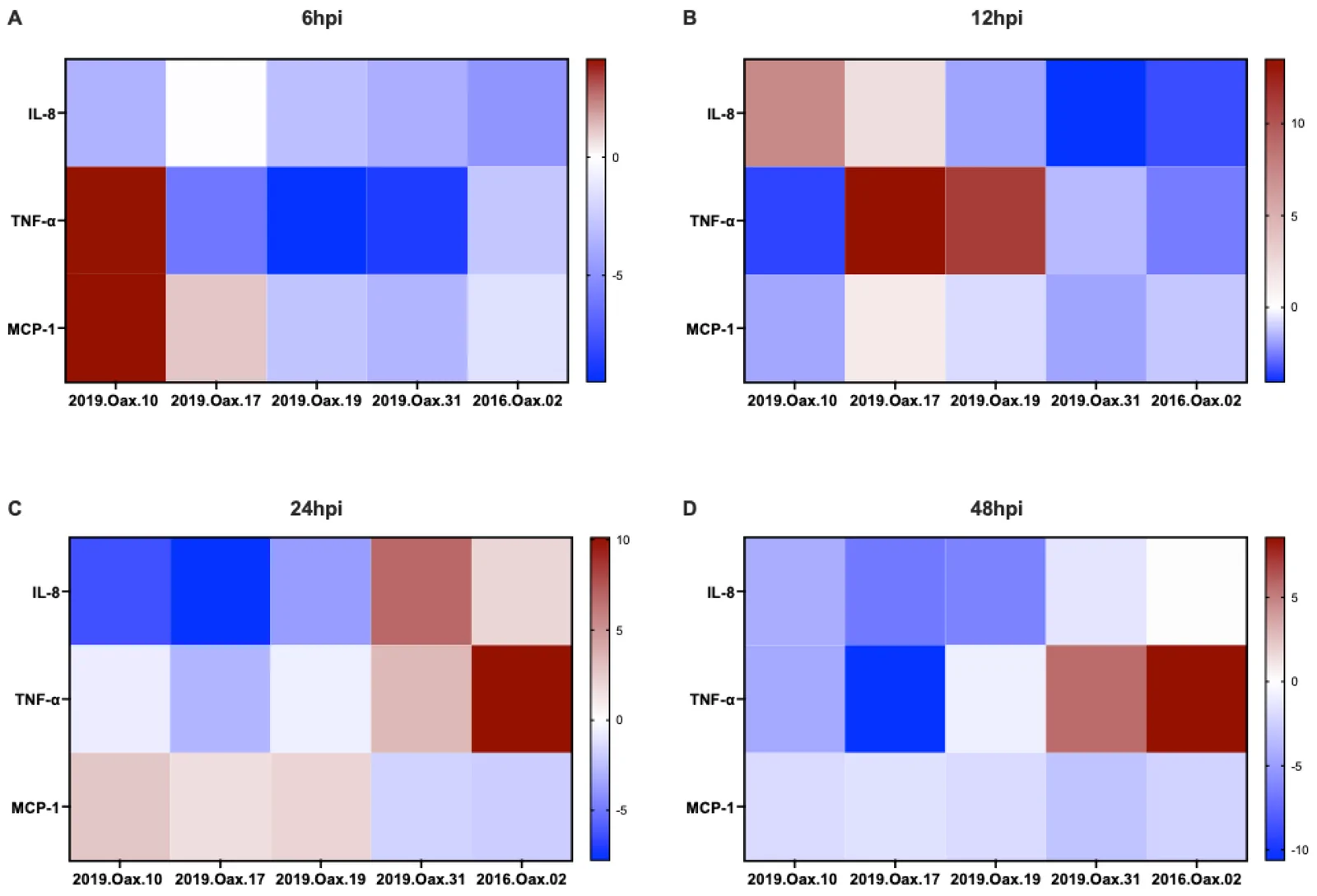
Clinical orthoflavivirus isolates exhibit heterogeneus inflammatory transcriptional responses in human keratinocytes. Relative expression of the inflammatory genes TNF-α, IL-8, and MCP-1 was determined by relative RT-qPCR in HaCaT cells infected with the five clinical orthoflavivirus isolates at a multiplicity of infection (MOI) of 1. Gene expression was evaluated at 6 h post-infection (hpi) (**A**), 12 hpi (**B**), 24 hpi (**C**), and 48 hpi (**D**). Relative expression levels were calculated using the 2^−ΔΔCt^ method with GAPDH as the endogenous reference gene and mock-infected cells as calibrators. Expression values are presented as log_2_ (2^−ΔΔCt^) obtained from three independent biological experiments. Red indicates relative upregulation, whereas blue indicates relative downregulation compared with non-infected controls. The individual biological replicate values are provided in Supplementary Table S2, whereas the complete statistical analyses are available in Supplementary Table S3.

To characterize the inflammatory response elicited by the five clinical orthoflavivirus isolates, the transcriptional expression of TNF-α, IL-8, and MCP-1 was evaluated by RT-qPCR at 6, 12, 24, and 48 h post-infection (hpi) (Figure 3).

Distinct temporal inflammatory transcriptional profiles were observed throughout the course of infection. At 6 hpi, TNF-α expression was markedly increased in isolate 2019.Oax.10, whereas comparatively lower transcript levels were detected in the remaining isolates (Figure 3A). In contrast, IL-8 expression showed only modest variation at this early time point, whereas MCP-1 exhibited elevated expression in 2019.Oax.10 and intermediate levels in the remaining isolates.

At 12 hpi, inflammatory gene-expression patterns became more diverse (Figure 3B). TNF-α transcript levels increased in isolates 2019.Oax.17 and 2019.Oax.19, whereas IL-8 expression was predominantly induced in 2019.Oax.10 and 2019.Oax.17. MCP-1 expression remained comparatively moderate across all isolates, although differences among isolates persisted.

By 24 hpi, inflammatory responses exhibited distinct temporal changes (Figure 3C). IL-8 expression reached its highest levels in 2019.Oax.31, whereas TNF-α expression was most pronounced in 2016.Oax.02. In contrast, MCP-1 displayed relatively homogeneous expression, with only moderate variation in transcript levels.

At 48 hpi, differences in inflammatory gene expression remained evident (Figure 3D). TNF-α expression remained elevated in 2019.Oax.31 and 2016.Oax.02, whereas IL-8expression was comparatively higher in 2016.Oax.02. MCP-1 showed limited variation at this late stage of infection, maintaining relatively similar transcript levels across the analyzed isolates.

To determine whether these transcriptional responses were accompanied by activation of the canonical inflammatory signaling pathway, phosphorylation of NF-κB p65 was evaluated by quantitative immunofluorescence (Figure 5). Representative fluorescence micrographs revealed detectable phosphorylated NF-κB p65 (p-p65) staining in infected cultures (Figure 5A), whereas lipopolysaccharide (LPS)-treated cells served as positive controls for pathway activation. Quantitative analysis based on automated whole-slide image segmentation and fluorescence-intensity measurements showed that all clinical isolates induced NF-κB activation relative to basal controls; however, the magnitude of p-p65 phosphorylation differed significantly among isolates (Figure 5B). Fluorescence-intensity measurements were obtained from all segmented cells within each whole-slide image, providing an unbiased assessment of signaling activity across the entire scanned specimen.

Overall, the combined transcriptional and quantitative immunofluorescence analyses indicate that the analyzed clinical orthoflavivirus isolates exhibit distinct inflammatory response profiles. The NF-κB phosphorylation analyses provide an independent functional assessment of inflammatory signaling that complements the longitudinal transcriptional analyses. Because phosphorylation was measured only at 12 h post-infection, these data represent an early signaling snapshot and should not be interpreted as temporal validation of the transcriptional responses. The individual biological replicate RT-qPCR values underlying the inflammatory gene-expression analyses (TNF-α, IL-8, MCP-1, and RANTES) are provided in Supplementary Table S2. Complete statistical results, including the two-way ANOVA followed by Tukey’s multiple-comparisons test, are presented in Supplementary Table S3.

### 3.4. Viral Replication and Host Transcriptional Responses Show No Consistent Association Across Clinical Orthoflavivirus Isolates

To determine whether viral replication was associated with the magnitude of innate immune gene expression, scatter plot analyses were performed by comparing infectious viral titers at 24 h post-infection (hpi) with representative antiviral and inflammatory transcriptional markers, including Mx1, IFN-β, RANTES, TNF-α, and IL-8 (Supplementary Figure S1).

Across the five clinical orthoflavivirus isolates, viral titers did not exhibit a consistent relationship with antiviral or inflammatory gene expression. Isolates with comparable infectious virus production displayed divergent transcriptional responses, whereas isolates with higher viral titers did not consistently exhibit greater induction of interferon-associated or inflammatory genes.

Spearman’s rank correlation analysis did not identify statistically significant associations between infectious viral titers and the selected host-response markers evaluated (Supplementary Figure S1). Given the limited number of isolates included in this study, these analyses were considered exploratory and were intended to identify overall trends rather than establish predictive relationships.

Collectively, these exploratory analyses indicate that, within this limited panel of clinical isolates, viral replication alone was not consistently associated with the magnitude of host innate immune activation. Accordingly, these observations should be interpreted cautiously and require validation using a larger and more diverse collection of clinical isolates.

### 3.5. Exploratory Integrated Visualization of Viral Replication and Transcriptional Responses Reveals Variable Phenotypic Profiles Among the Analyzed Clinical Orthoflavivirus Isolates

**Figure 4:**
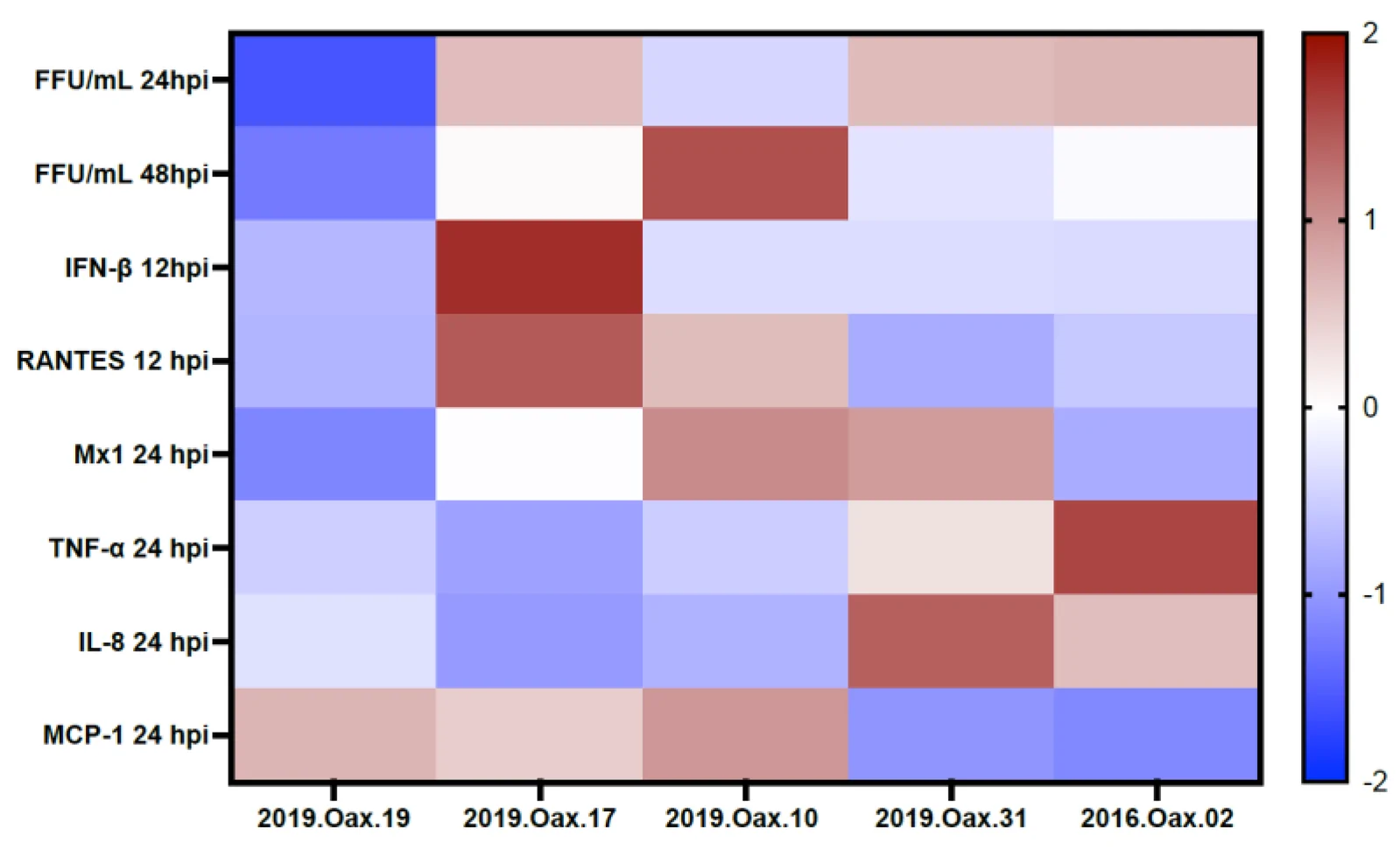
Exploratory integrated visualization of viral replication and host transcriptional responses across clinical orthoflavivirus isolates.

To obtain an integrated overview of the phenotypic diversity exhibited by the five clinical orthoflavivirus isolates, viral replication and host transcriptional datasets were combined using row-wise Z-score normalization (Figure 4). The integrated analysis included infectious viral titers at 24 and 48 hpi, representative antiviral genes (IFN-β, Mx1, and RANTES), and inflammatory mediators (TNF-α, IL-8, and MCP-1).

The resulting heatmap provides an exploratory visual summary of the combined virological and transcriptional datasets. Differences among isolates are presented descriptively based on manually ordered phenotypic profiles rather than an unsupervised clustering approach. Rather than segregating according to viral replication alone, the isolates differed in the temporal balance between antiviral and inflammatory transcriptional responses, indicating that host-response profiles represent an additional dimension of phenotypic variability.

Isolates with comparable viral titers frequently exhibited distinct transcriptional signatures, whereas isolates displaying similar antiviral gene-expression patterns differed in the magnitude of their inflammatory responses. These observations are consistent with the correlation analyses (Supplementary Figure S1), which indicate that viral replication was not consistently associated with the intensity of host innate immune activation.

Overall, this exploratory integrative visualization facilitates descriptive comparison of viral replication and host transcriptional responses across the analyzed clinical isolates rather than providing a formal multivariate classification. Instead, the heatmap summarizes the multidimensional phenotypic characteristics observed in this study. This integrative approach highlights the functional heterogeneity of the analyzed clinical orthoflavivirus isolates and provides a useful framework for comparative characterization of virus–host interactions.

Heatmap providing an exploratory descriptive summary of viral replication and host transcriptional responses across the analyzed isolates. The analysis included infectious viral titers at 24 and 48 h post-infection (hpi), antiviral genes (IFN-β, Mx1, and RANTES), and inflammatory genes (TNF-α, IL-8, and MCP-1). Data were standardized using row-wise Z-score normalization to facilitate visualization. Red indicates values above the mean for each variable, white indicates values close to the mean, and blue indicates values below the mean. The heatmap illustrates characteristic combinations of viral replication and innate immune transcriptional responses. No clustering algorithm was applied, and isolates were manually ordered according to their phenotypic profiles to facilitate biological interpretation. Therefore, the figure should be interpreted as a descriptive synthesis rather than as a formal multivariate classification.

### 3.6. Quantitative Whole-Slide Immunofluorescence Validates Differences in STAT1 and NF-κB Signaling Activation Among the Analyzed Clinical Orthoflavivirus Isolates

**Figure 5:**
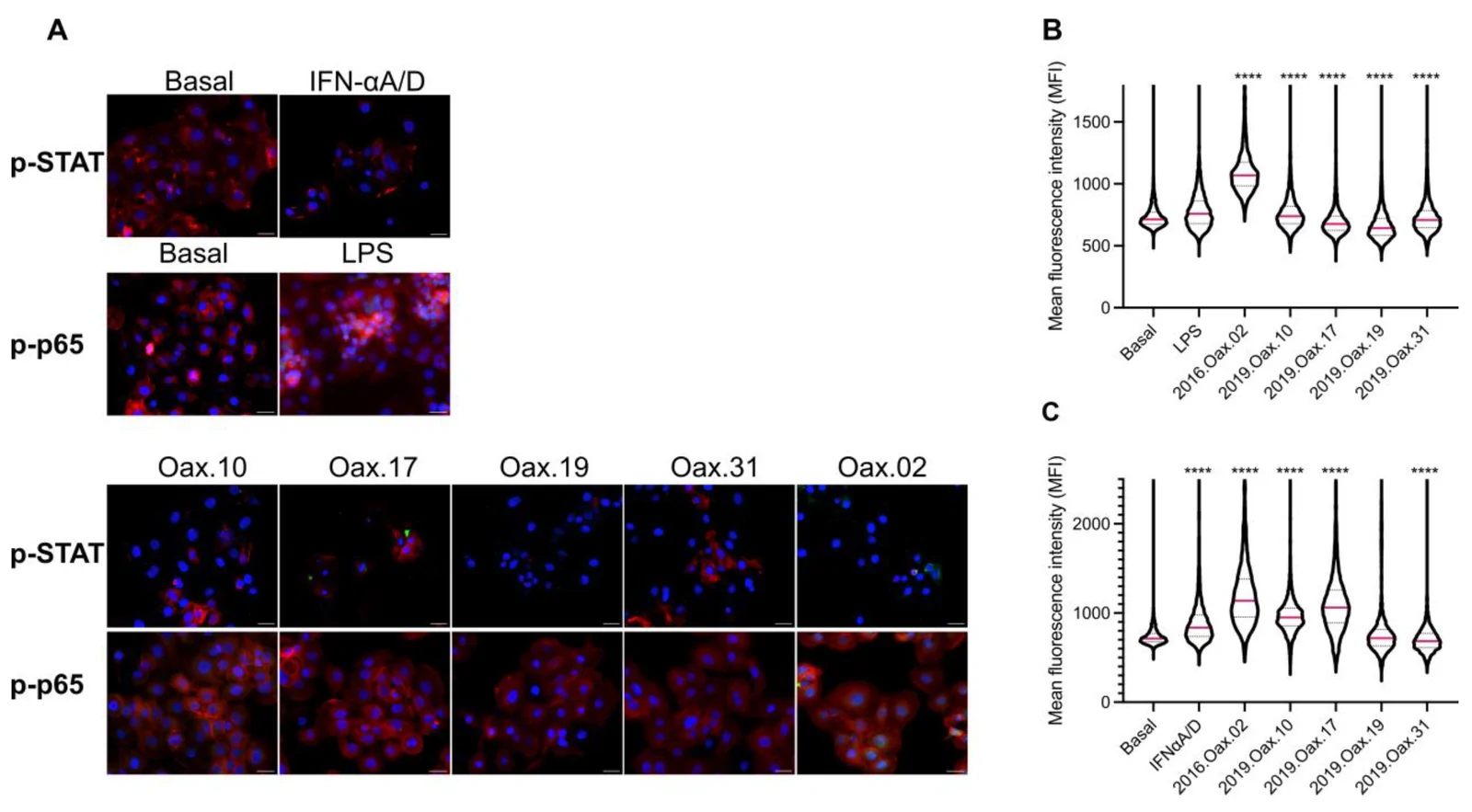
Validation of STAT1 and NF-κB activation by quantitative immunofluorescence in human keratinocytes infected with clinical orthoflavivirus isolates. (**A**) Representative immunofluorescence images of HaCaT cells infected with the five clinical orthoflavivirus isolates at a multiplicity of infection (MOI) of 1 for 12 h post-infection (hpi), showing phosphorylated STAT1 (p-STAT1, Tyr701) and phosphorylated NF-κB p65 (p-p65, Ser536). Nuclei were counterstained with DAPI. Recombinant human IFN-α A/D (100 IU/mL for 20 min) was included as a positive control for STAT1 phosphorylation, whereas lipopolysaccharide (LPS; 1 µg/mL for 2 h) was included as a positive control for NF-κB p65 phosphorylation. Positive-control samples were processed in parallel with infected cultures under identical staining and imaging conditions. Scale bar = 20 µm. (**B**) Quantification of p-p65 fluorescence intensity obtained by automated whole-slide single-cell image analysis. (**C**) Quantification of p-STAT1 fluorescence intensity obtained by automated whole-slide single-cell image analysis. Whole-slide images were acquired using the Zeiss Axio Scan.Z1 and analyzed using an automated segmentation pipeline. Fluorescence intensity was quantified for all segmented cells within each whole-slide image. For each biological replicate, the median fluorescence intensity (MFI) across all segmented cells was calculated to generate a single representative value per replicate (*n* = 3 independent biological replicates per condition). Statistical significance was determined using the Kruskal–Wallis test followed by Dunn’s multiple-comparisons test based on these replicate-level median MFI values. Violin plots display the distribution of single-cell MFI values pooled from the three independent biological replicates for visualization purposes only and were not used for statistical inference. The horizontal pink line indicates the median single-cell fluorescence intensity, whereas the gray dotted lines indicate the first and third quartiles. Brackets denote the pairwise comparisons evaluated by Dunn’s multiple-comparisons test. Statistical significance is indicated as follows: *p* < 0.0001 (****). Replicate-level median fluorescence intensity values and the corresponding statistical comparisons are provided in Supplementary Figure S3 and Tables S4 and S5.

To determine whether the transcriptional differences observed among the clinical orthoflavivirus isolates were accompanied by activation of key innate immune signaling pathways, phosphorylation of STAT1 (Tyr701) and NF-κB p65 (Ser536) was evaluated by quantitative immunofluorescence using an automated whole-slide single-cell image-analysis workflow (Figure 5).

Representative fluorescence micrographs revealed distinct patterns of p-STAT1 and phosphorylated NF-κB p65 (p-p65) staining among infected cultures (Figure 5A). Recombinant human IFN-α A/D-treated cells and lipopolysaccharide (LPS)-treated cells were included as positive controls for STAT1 and NF-κB activation, respectively, whereas mock-infected cultures served as basal controls. Whole-slide images were segmented using an automated single-cell analysis pipeline, allowing fluorescence intensity to be quantified for every segmented cell within each biological replicate. Statistical comparisons were performed using the median mean fluorescence intensity (MFI) obtained from each biological replicate (*n* = 3), whereas pooled single-cell fluorescence-intensity values were used exclusively to visualize fluorescence distributions in the violin plots.

Quantitative analysis demonstrated that all five clinical orthoflavivirus isolates induced detectable phosphorylation of both STAT1 and NF-κB p65 relative to basal controls, although the magnitude of activation differed significantly among isolates (Figure 5B,C).

The heterogeneous patterns of STAT1 phosphorylation were consistent with the antiviral transcriptional profiles described in Figure 2, whereas differences in NF-κB p65 activation paralleled the inflammatory transcriptional responses presented in Figure 3. Together, these findings provide functional evidence that clinical orthoflavivirus isolates differentially modulate key innate immune signaling pathways at both the transcriptional and protein levels.

## 4. Discussion

Orthoflaviviruses comprise genetically diverse mosquito-borne pathogens responsible for a broad spectrum of clinical manifestations, yet the functional consequences of intra- and interspecies viral diversity on early host responses remain incompletely understood. In the present study, we performed an exploratory integrated phenotypic characterization of five clinical orthoflavivirus isolates obtained in Oaxaca, Mexico, by combining virological, transcriptional, and quantitative imaging approaches. Although all isolates established productive infection in human keratinocytes, they exhibited distinct infection phenotypes, viral replication profiles, and innate immune responses, demonstrating substantial functional heterogeneity despite identical experimental conditions.

All five isolates efficiently infected HaCaT cells, confirming that human keratinocytes constitute a permissive epithelial model for early orthoflavivirus infection, consistent with previous reports [15]. Differences in immunostained focus appearance, infection patterns, and infectious virus production indicated phenotypic variability among isolates. However, differences in viral amplification were not uniformly distributed across the isolate panel. Instead, statistically significant differences were detected only between selected isolate pairs, indicating partially overlapping replication profiles rather than discrete high- and low-replicating phenotypes. These observations suggest that viral replication represents only one component of the functional diversity observed among the analyzed isolates.

The greatest variability was observed in antiviral transcriptional responses. Type I interferon-associated genes, including IFN-β, Mx1, OAS1, PKR, IFITM3, Viperin, and RANTES, displayed distinct temporal expression profiles throughout infection. Differences were evident not only in the magnitude of gene induction but also in the timing of activation, indicating variation in the kinetics of antiviral signaling among isolates. These findings are consistent with previous studies demonstrating that orthoflaviviruses employ multiple strategies to modulate interferon production and signaling through viral proteins that antagonize pattern-recognition receptor pathways, JAK–STAT signaling, and interferon-stimulated gene expression [16]. Our data further suggest that the analyzed isolates differed substantially in their capacity to engage these antiviral pathways despite establishing productive infection.

Inflammatory responses exhibited a similarly heterogeneous pattern. Expression of TNF-α, IL-8, and MCP-1 varied considerably among isolates throughout the infection time course, indicating dynamic differences in inflammatory pathway activation. Because excessive inflammatory responses have been implicated in vascular permeability, tissue damage, and disease severity during several orthoflavivirus infections, these differences may have biological relevance beyond viral replication alone [17]. Although the present study was not designed to evaluate pathogenicity, the observed transcriptional diversity suggests that naturally circulating clinical isolates may differentially influence early epithelial inflammatory responses that contribute to downstream immune activation.

Importantly, quantitative whole-slide immunofluorescence provided functional validation of the transcriptional findings. Automated single-cell image analysis was consistent with the observed differences in phosphorylation of both STAT1 and NF-κB p65, providing an independent functional assessment of innate immune signaling that complemented the transcriptional analyses. Because these analyses were performed independently of the transcriptional profiling and at a single early infection time point, they provide complementary evidence that the observed differences extend beyond mRNA abundance and are reflected at the level of intracellular signaling. Furthermore, automated whole-slide image analysis minimized observer bias and enabled quantitative assessment of signaling activity across entire cell populations rather than manually selected microscopic fields.

Another exploratory observation of this study was the absence of a consistent relationship between infectious viral titers and the magnitude of antiviral or inflammatory gene expression. Although the limited number of clinical isolates precluded definitive correlation analyses, isolates with comparable infectious virus production frequently exhibited markedly different transcriptional profiles, whereas isolates displaying similar antiviral responses differed in viral amplification. Collectively, these findings indicate that, within this limited dataset, viral burden alone was not consistently associated with the host-response markers evaluated. These observations are consistent with the functional diversity observed among the analyzed isolates being influenced by multiple factors, including viral sensing, downstream signaling pathways, viral replication dynamics, and potentially differences in the proportion of infected and bystander cells. However, the present exploratory study was not designed to distinguish the relative contribution of these mechanisms. The present study was not designed to distinguish the relative contribution of these mechanisms.

This study has several limitations. First, only five clinical isolates were analyzed, and all experiments were performed in a single epithelial cell line under in vitro conditions. Consequently, the correlation analyses should be considered exploratory and should not be interpreted as evidence for the absence of biological associations between viral replication and host-response markers. Second, the integrated heatmap analysis was based exclusively on viral replication and transcriptional datasets, whereas quantitative immunofluorescence was evaluated independently at a single early infection time point (12 hpi) as a complementary functional assessment of signaling-pathway activation rather than as temporal validation of the transcriptional responses.

The isolate panel comprised representatives of three orthoflavivirus species (DENV-2, WNV, and ZIKV), including two ZIKV isolates, two WNV isolates, and one DENV-2 isolate. Because the isolates were selected from a larger institutional collection according to predefined laboratory criteria rather than clinical outcome, the study was designed to compare the functional behavior of representative clinical isolates rather than establish associations with disease severity. Consequently, although clear functional phenotypic differences were observed, the experimental design does not allow isolate-specific effects to be completely distinguished from species-specific biological characteristics. Moreover, although viral replication kinetics were independently quantified in HaCaT cells and demonstrated distinct replication profiles among the analyzed isolates, the proportion of infected cells was not directly quantified at the corresponding transcriptional sampling time points. Therefore, the observed transcriptional and signaling responses likely reflect a combination of intrinsic virus–host interactions together with viral replication dynamics, infection frequency, and paracrine signaling. Accordingly, the present experimental design does not allow the relative contribution of these factors to be distinguished. Future studies including larger numbers of isolates from each viral species, together with viral genomic analyses, quantitative assessment of infection frequency, additional target cell types, and in vivo models, will be necessary to distinguish isolate-level from species-level variation and to define the molecular determinants underlying differential innate immune modulation.

Finally, although GAPDH Ct values remained stable across all experimental conditions evaluated, supporting its suitability as the endogenous reference gene for relative quantification, a formal assessment of reference gene stability using dedicated algorithms (e.g., geNorm or NormFinder) was not performed. Accordingly, the possibility of subtle normalization bias cannot be completely excluded.

Overall, our findings suggest that naturally circulating clinical orthoflavivirus isolates exhibit substantial functional heterogeneity in which differences in viral replication alone do not consistently account for the observed host-response profiles within this isolate panel. The integrated evaluation of viral replication, host transcriptional responses, and complementary quantitative signaling analyses indicates that each isolate exhibits a distinct innate immune response profile under identical experimental conditions. Although the present study does not establish the direction of causality between viral replication and innate immune activation, the combined datasets demonstrate that viral burden alone does not fully account for the observed biological diversity. Together, these findings highlight the value of integrating functional characterization with conventional virological analyses to improve our understanding of orthoflavivirus biology and provide a framework for investigating how naturally occurring viral diversity shapes virus–host interactions and disease pathogenesis.

## 5. Conclusions

This study reveals that the panel of clinical orthoflavivirus isolates collected in Oaxaca, Mexico, exhibited substantial functional heterogeneity despite establishing productive infection under identical experimental conditions. Although all isolates successfully infected human keratinocytes, they displayed distinct viral replication profiles together with markedly different antiviral and inflammatory transcriptional responses. Within this limited panel of clinical isolates, host innate immune activation was not consistently associated with infectious viral production, suggesting that viral burden alone does not fully account for the observed diversity of host-response profiles. Although the present experimental design does not allow the relative contribution of viral replication dynamics, infection frequency, and innate immune activation to be distinguished, the findings are consistent with multiple virus–host interactions contributing to the observed functional diversity.

The exploratory integration of virological, transcriptional, and quantitative imaging datasets identified distinct innate immune response profiles among the analyzed isolates, whereas quantitative whole-slide immunofluorescence provided an independent functional assessment of STAT1 and NF-κB signaling activation that complemented the transcriptional analyses. Together, these findings indicate that the analyzed orthoflavivirus isolates exhibit distinct host-response profiles at both the transcriptional and signaling levels under identical experimental conditions.

Overall, our findings highlight the value of integrated functional phenotyping for improving our understanding of the biological diversity of clinical orthoflavivirus isolates. Although exploratory in nature, this integrative framework provides a foundation for future studies incorporating larger isolate collections, quantitative assessment of infection frequency, viral genomic analyses, and additional experimental models to define how naturally occurring viral diversity shapes virus–host interactions, immune modulation, viral transmission, and disease pathogenesis.

## Figures and Tables

**Table 1:** Origin, identification, and passage history of the clinical orthoflavivirus isolates used in this study.

Isolate	Virus Species	Year of Collection	Clinical Sample	Selection Criteria	Passage Used
2019.Oax.10	ZIKV	2019	Serum	Representative isolate with confirmed RT-PCR and successful isolation	P3
2019.Oax.17	WNV	2019	Serum	Representative isolate with confirmed RT-PCR and successful isolation	P3
2019.Oax.19	ZIKV	2019	Serum	Representative isolate with confirmed RT-PCR and successful isolation	P3
2019.Oax.31	DENV-2	2019	Serum	Representative isolate with confirmed RT-PCR and successful isolation	P3
2016.Oax.02	WNV	2016	Serum	Representative isolate with confirmed RT-PCR and successful isolation	P3

Abbreviations: RT-PCR, reverse transcription polymerase chain reaction; P3, third cell-culture passage. All viral isolates were molecularly confirmed prior to experimentation and propagated under standardized laboratory conditions. Experimental infections were performed using low-passage viral stocks (P3) to minimize phenotypic changes associated with in vitro adaptation.

## Data Availability

The data supporting the findings of this study are available from the corresponding author upon reasonable request.

## Supplementary Materials

The following supporting information can be downloaded at: https://www.mdpi.com/article/10.3390/v18080826/s1. Table S1: RT-qPCR primer sequences, amplification conditions, primer sources, and corresponding references or catalog numbers used in this study; Table S2: Individual biological replicate values, means, and standard deviations for the RT-qPCR analyses presented in Figure 2 and Figure 3; Table S3: Quantitative fluorescence intensity values obtained from whole-slide immunofluorescence analyses; Table S4: Pairwise statistical comparisons of phosphorylated STAT1 median fluorescence intensity, including mean differences, 95% confidence intervals, and exact unadjusted *p*-values; Table S5: Pairwise statistical comparisons of phosphorylated NF-κB p65 median fluorescence intensity, including mean differences, 95% confidence intervals, and exact unadjusted *p*-values; Figure S1: Exploratory correlation analysis between viral replication and host innate immune gene expression; Figure S2: Integrated heatmap summarizing viral replication kinetics and transcriptional responses across the analyzed orthoflavivirus isolates; Figure S3: Biological replicate-level median fluorescence intensity values for phosphorylated STAT1 (A) and phosphorylated NF-κB p65 (B).

## Abbreviations

The following abbreviations are used in this manuscript:

Abbreviation	Definition
Ct	Cycle Threshold
DAPI	4′,6-Diamidino-2-Phenylindole
DENV	Dengue Virus
FFU	Focus-Forming Units
FFU/mL	Focus-Forming Units per Milliliter
GAPDH	Glyceraldehyde-3-Phosphate Dehydrogenase
hpi	Hours Post-Infection
IFN-I	Type I Interferon
IFNαA/D	hybrid recombinant human interferon-αA/human interferon-αD
IFNβ	Interferon Beta
IFITM3	Interferon-Induced Transmembrane Protein 3
IL-8	Interleukin 8
ISGs	Interferon-Stimulated Genes
JAK	Janus Kinase
LPS	Lipopolysaccharide
MCP-1	Monocyte Chemoattractant Protein-1
MOI	Multiplicity of Infection
Mx1	MX Dynamin Like GTPase 1
NF-κB	Nuclear Factor Kappa B
OAS1	2′-5′-Oligoadenylate Synthetase 1
p-p65	Phosphorylated NF-κB p65
p-STAT1	Phosphorylated Signal Transducer and Activator of Transcription 1
PBS	Phosphate-Buffered Saline
PCR	Polymerase Chain Reaction
PKR	Protein Kinase R
qPCR	Quantitative Polymerase Chain Reaction
RANTES	Regulated upon Activation, Normal T Cell Expressed and Secreted
RNA	Ribonucleic Acid
RT	Reverse Transcription
RT-qPCR	Reverse Transcription Quantitative Polymerase Chain Reaction
SD	Standard Deviation
STAT1	Signal Transducer and Activator of Transcription 1
TNF-α	Tumor Necrosis Factor Alpha
WNV	West Nile Virus
ZIKV	Zika Virus
