# Supplementary material for: Integrated Functional Characterization of a Panel of Clinical Orthoflavivirus Isolates Reveals Distinct Replication and Innate Immune Response Profiles in Human Keratinocytes
Source: Viruses. 2026 Jul 27;18(8):826. doi: 10.3390/v18080826 (PMC13517277; doi:10.3390/v18080826)
Supplement: Supplementary file 1 [file viruses-18-00826-s001.zip › viruses-4438188-supplementary.pdf]

**Table S1. Primers and RT-PCR amplification conditions used in this study.**

| Gene | Forward primer (5′→3′) | Reverse primer (5′→3′) | Annealing temperature (°C) | Primer sequence source | Reference /Catalog No. |
|---|---|---|---|---|---|
| **IFNβ1** | CTTGGATTCCTACAAAGAAGCAGC | TCCTCCTTCTGGAACTGCTGCA | 60 | OriGene Technologies | HP205913 |
| **MX1** | GGCTGTTTACCAGACTCCGACA | CACAAAGCCTGGCAGCTCTCTA | 60 | OriGene Technologies | HP206142 |
| **OAS1** | AGGAAAGGTGCTTCCGAGGTAG | GGACTGAGGAAGACAACCAGGT | 60 | OriGene Technologies | HP234311 |
| **PKR** | GAAGTGGACCTCTACGCTTTGG | TGATGCCATCCCGTAGGTCTGT | 60 | OriGene Technologies | HP206392 |
| **IFITM3** | CTGGGCTTCATAGCATTCGCCT | AGATGTTCAGGCACTTGGCGGT | 60 | OriGene Technologies | HP214009 |
| **Viperin** | CCAGTGCAACTACAAATGCGGC | CGGTCTTGAAGAAATGGCTCTCC | 60 | OriGene Technologies | HP216708 |
| **GAPDH** | GTGAAGGTCGGAGTCAACGGAT | GCCAGCATCGCCCCACTTG | 57 | Published protocol | [14] |
| **TNF** | GGCTCCAGGCGGTGCTTGTTC | AGACGGCGATGCGGCTGATG | 56 | Published protocol | [12] |
| **IL8** | CAGTTTTGCCAAGGAGTGCTAA | AACTTCTCCACAACCCTCTGC | 57 | Published protocol | [11] |
| **MCP-1** | GATCTCAGTGCAGAGGCTCG | TGCTTGTCCAGGTGGTCCAT | 65 | Published protocol | [13] |
| **RANTES** | ACCACACCCTGCTGCTTTGC | CCG AAC CCA TTT CTT CTC TGG | 55 | Published protocol | [13] |

Notes: Primer sequences for IFNB1, MX1, OAS1, PKR, IFITM3, and RSAD2 (Viperin) were obtained from commercially available human RT-qPCR primer sets (OriGene Technologies); the corresponding catalog numbers are provided in the Reference/Catalog No. column. Primer sequences for GAPDH, TNF, IL8, MCP-1, and RANTES were adopted from previously published RT-qPCR protocols, and the corresponding references are indicated in the same column.

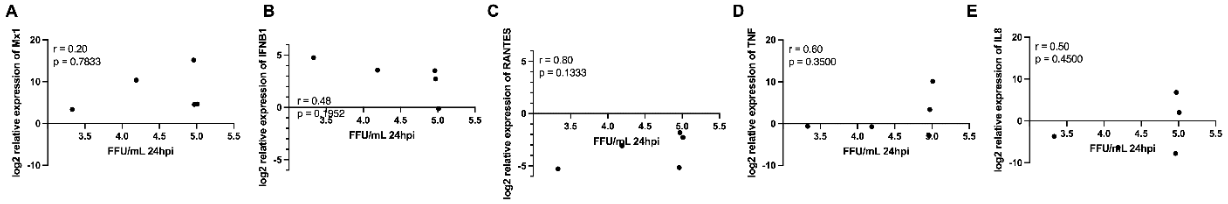


**Supplementary Figure S1. Correlation between viral replication and host innate immune gene expression.** Scatter plots showing Spearman correlation analyses between infectious viral titers ($log_{10}$ FFU/mL) and representative antiviral (IFNB1, Mx1, and CCL5/RANTES) and inflammatory (TNF and IL8) gene expression measured by relative RT-qPCR. No statistically

significant correlations were detected between viral replication and the selected transcriptional or signaling markers within this limited panel of clinical orthoflavivirus isolates. Because of the exploratory nature of the analysis and the small number of isolates evaluated, these findings should not be interpreted as evidence of independence between these variables.

**Supplementary Table S2. Individual biological replicate values used for RT-qPCR analysis**

| Gene | Condition | Time (hpi) | Rep 1 $log_2FC$ | Rep 2 $log_2FC$ | Rep 3 $log_2FC$ | Mean $log_2FC$ | SD |
|---|---|---|---|---|---|---|---|
| **OAS1** | NI | 24 | 0 | 0 | 0 | 0 | 0 |
| **OAS1** | IFN-α A/D 10 IU | 6 | -5.63 | -4.66 | -5.20 | -5.16 | 0.49 |
| **OAS1** | IFN-α A/D 10 IU | 12 | 4.71 | 4.85 | 5.10 | 4.89 | 0.2 |
| **OAS1** | IFN-α A/D 10 IU | 24 | -2.27 | -1.88 | -2.10 | -2.08 | 0.2 |
| **OAS1** | IFN-α A/D 10 IU | 48 | 2.18 | 2.60 | 2.70 | 2.49 | 0.28 |
| **OAS1** | IFN-α A/D 100 IU | 6 | -0.50 | -0.51 | -0.40 | -0.47 | 0.06 |
| **OAS1** | IFN-α A/D 100 IU | 12 | 1.68 | 1.79 | 1.72 | 1.73 | 0.06 |
| **OAS1** | IFN-α A/D 100 IU | 24 | -1.59 | -1.38 | -1.41 | -1.46 | 0.11 |
| **OAS1** | IFN-α A/D 100 IU | 48 | 7.48 | 7.31 | 7.40 | 7.4 | 0.09 |
| **OAS1** | 2019.Oax.10 | 6 | -7.09 | -7.69 | -7.30 | -7.36 | 0.3 |
| **OAS1** | 2019.Oax.10 | 12 | -2.29 | -2.55 | -2.30 | -2.38 | 0.15 |
| **OAS1** | 2019.Oax.10 | 24 | -3.28 | -3.47 | -3.40 | -3.38 | 0.1 |
| **OAS1** | 2019.Oax.10 | 48 | 1.57 | 1.48 | 1.40 | 1.48 | 0.09 |
| **OAS1** | 2019.Oax.17 | 6 | -3.84 | -3.37 | -3.80 | -3.67 | 0.26 |
| **OAS1** | 2019.Oax.17 | 12 | -1.07 | -1.10 | -1.30 | -1.16 | 0.13 |
| **OAS1** | 2019.Oax.17 | 24 | -4.91 | -3.28 | -3.60 | -3.93 | 0.86 |
| **OAS1** | 2019.Oax.17 | 48 | 0.87 | 0.82 | 0.86 | 0.85 | 0.03 |
| **OAS1** | 2019.Oax.19 | 6 | -8.37 | -6.32 | -8.46 | -7.72 | 1.21 |
| **OAS1** | 2019.Oax.19 | 12 | -4.12 | -4.20 | -4.16 | -4.16 | 0.04 |
| **OAS1** | 2019.Oax.19 | 24 | -4.39 | -4.60 | -4.52 | -4.5 | 0.11 |
| **OAS1** | 2019.Oax.19 | 48 | -0.65 | -0.69 | -0.67 | -0.67 | 0.02 |
| **OAS1** | 2019.Oax.31 | 6 | -7.56 | -7.64 | -7.6 | -7.6 | 0.04 |
| **OAS1** | 2019.Oax.31 | 12 | -4.95 | -5.24 | -5.09 | -5.09 | 0.15 |
| **OAS1** | 2019.Oax.31 | 24 | -3.23 | -3.65 | -3.47 | -3.45 | 0.21 |
| **OAS1** | 2019.Oax.31 | 48 | -0.50 | -0.28 | -0.4 | -0.39 | 0.11 |
| **OAS1** | 2016.Oax.02 | 6 | -9.04 | -10.14 | -9.82 | -9.67 | 0.57 |
| **OAS1** | 2016.Oax.02 | 12 | -3.42 | -4.43 | -4.13 | -3.99 | 0.52 |
| **OAS1** | 2016.Oax.02 | 24 | -4.35 | -4.31 | -4.33 | -4.33 | 0.02 |
| **OAS1** | 2016.Oax.02 | 48 | -0.19 | -0.42 | -0.33 | -0.31 | 0.12 |

**Supplementary Table S2. (Continued)**

| Gene | Condition | Time (hpi) | Rep 1 $log_2FC$ | Rep 2 $log_2FC$ | Rep 3 $log_2FC$ | Mean $log_2FC$ | SD |
|---|---|---|---|---|---|---|---|
| **PKR** | NI | 24 | 0 | 0 | 0 | 0 | 0 |
| **PKR** | IFN-α A/D 10 IU | 6 | 0.92 | 0.31 | 0.61 | 0.61 | 0.31 |
| **PKR** | IFN-α A/D 10 IU | 12 | 9.32 | 9.49 | 9.41 | 9.41 | 0.09 |
| **PKR** | IFN-α A/D 10 IU | 24 | -1.06 | -0.5 | -0.78 | -0.78 | 0.28 |
| **PKR** | IFN-α A/D 10 IU | 48 | 9.34 | 8.4 | 8.87 | 8.87 | 0.47 |
| **PKR** | IFN-α A/D 100 IU | 6 | 0.78 | 0.36 | 0.57 | 0.57 | 0.21 |
| **PKR** | IFN-α A/D 100 IU | 12 | 3.45 | 3.91 | 3.68 | 3.68 | 0.23 |
| **PKR** | IFN-α A/D 100 IU | 24 | -0.75 | 0.02 | -0.37 | -0.37 | 0.39 |
| **PKR** | IFN-α A/D 100 IU | 48 | 8.68 | 8.93 | 8.8 | 8.8 | 0.13 |
| **PKR** | 2019.Oax.10 | 6 | -0.77 | -0.91 | -0.84 | -0.84 | 0.07 |
| **PKR** | 2019.Oax.10 | 12 | 3.83 | 3.66 | 3.74 | 3.74 | 0.08 |
| **PKR** | 2019.Oax.10 | 24 | -1.03 | -0.74 | -0.89 | -0.89 | 0.15 |
| **PKR** | 2019.Oax.10 | 48 | 0.54 | 0.33 | 0.43 | 0.43 | 0.11 |
| **PKR** | 2019.Oax.17 | 6 | -4.28 | -2.56 | -3.42 | -3.42 | 0.86 |
| **PKR** | 2019.Oax.17 | 12 | 3.85 | 3.95 | 3.9 | 3.9 | 0.05 |
| **PKR** | 2019.Oax.17 | 24 | -2.93 | -4.94 | -3.94 | -3.94 | 1.01 |
| **PKR** | 2019.Oax.17 | 48 | -0.47 | -0.29 | -0.38 | -0.38 | 0.09 |
| **PKR** | 2019.Oax.19 | 6 | -5.19 | -3.95 | -4.57 | -4.57 | 0.62 |
| **PKR** | 2019.Oax.19 | 12 | -0.64 | -1.35 | -0.99 | -0.99 | 0.35 |
| **PKR** | 2019.Oax.19 | 24 | -0.72 | -1.27 | -0.99 | -0.99 | 0.28 |
| **PKR** | 2019.Oax.19 | 48 | -2.2 | -0.87 | -1.53 | -1.53 | 0.67 |
| **PKR** | 2019.Oax.31 | 6 | -3.17 | -4.81 | -3.99 | -3.99 | 0.82 |
| **PKR** | 2019.Oax.31 | 12 | -1.31 | -0.69 | -1 | -1 | 0.31 |
| **PKR** | 2019.Oax.31 | 24 | -1.57 | -2.62 | -2.1 | -2.1 | 0.53 |
| **PKR** | 2019.Oax.31 | 48 | 0.85 | 0.18 | 0.51 | 0.51 | 0.34 |
| **PKR** | 2016.Oax.02 | 6 | -4.42 | -5.89 | -5.16 | -5.16 | 0.74 |
| **PKR** | 2016.Oax.02 | 12 | -0.19 | -0.16 | -0.17 | -0.17 | 0.02 |
| **PKR** | 2016.Oax.02 | 24 | -1.56 | -2.09 | -1.83 | -1.83 | 0.27 |
| **PKR** | 2016.Oax.02 | 48 | 0.8 | 0.59 | 0.69 | 0.69 | 0.11 |

**Supplementary Table S2. (Continued)**

| Gene | Condition | Time (hpi) | Rep 1 $log_2FC$ | Rep 2 $log_2FC$ | Rep 3 $log_2FC$ | Mean $log_2FC$ | SD |
|---|---|---|---|---|---|---|---|
| **Viperin** | NI | 24 | 0 | 0 | 0 | 0 | 0 |
| **Viperin** | IFN-α A/D 10 IU | 6 | -1.82 | -2.85 | -2.33 | -2.333 | 0.515 |
| **Viperin** | IFN-α A/D 10 IU | 12 | 0.12 | 0.421 | 0.27 | 0.27 | 0.15 |
| **Viperin** | IFN-α A/D 10 IU | 24 | -3.75 | -2.71 | -3.23 | -3.23 | 0.52 |
| **Viperin** | IFN-α A/D 10 IU | 48 | 1.06 | 0.64 | 0.85 | 0.85 | 0.21 |
| **Viperin** | IFN-α A/D 100 IU | 6 | 2.43 | 2.77 | 2.6 | 2.6 | 0.17 |
| **Viperin** | IFN-α A/D 100 IU | 12 | 2.409 | 2.685 | 2.55 | 2.548 | 0.138 |
| **Viperin** | IFN-α A/D 100 IU | 24 | -0.88 | -0.97 | -0.93 | -0.927 | 0.045 |
| **Viperin** | IFN-α A/D 100 IU | 48 | 5.32 | 5 | 5.16 | 5.16 | 0.16 |
| **Viperin** | 2019.Oax.10 | 6 | -1.02 | -0.56 | -0.79 | -0.79 | 0.23 |
| **Viperin** | 2019.Oax.10 | 12 | 5.767 | 5.771 | 5.77 | 5.769 | 0.002 |
| **Viperin** | 2019.Oax.10 | 24 | -5.2 | -4.48 | -4.84 | -4.84 | 0.36 |
| **Viperin** | 2019.Oax.10 | 48 | 5.01 | 5.03 | 5.02 | 5.02 | 0.01 |
| **Viperin** | 2019.Oax.17 | 6 | -6.94 | -6.7 | -6.82 | -6.82 | 0.12 |
| **Viperin** | 2019.Oax.17 | 12 | -2.074 | -3.19 | -2.63 | -2.631 | 0.558 |
| **Viperin** | 2019.Oax.17 | 24 | -7.36 | -5.6 | -6.48 | -6.48 | 0.88 |
| **Viperin** | 2019.Oax.17 | 48 | 4.07 | 3.95 | 4.01 | 4.01 | 0.06 |
| **Viperin** | 2019.Oax.19 | 6 | -8.38 | -8.41 | -8.39 | -8.393 | 0.015 |
| **Viperin** | 2019.Oax.19 | 12 | -5.319 | -6.256 | -5.79 | -5.788 | 0.469 |
| **Viperin** | 2019.Oax.19 | 24 | -5.37 | -4.43 | -4.9 | -4.9 | 0.47 |
| **Viperin** | 2019.Oax.19 | 48 | -0.84 | -0.16 | -0.5 | -0.5 | 0.34 |
| **Viperin** | 2019.Oax.31 | 6 | -7.93 | -5.91 | -6.92 | -6.92 | 1.01 |
| **Viperin** | 2019.Oax.31 | 12 | -9.201 | -7.206 | -8.2 | -8.202 | 0.998 |
| **Viperin** | 2019.Oax.31 | 24 | -5.759 | -5.023 | -5.39 | -5.39 | 0.368 |
| **Viperin** | 2019.Oax.31 | 48 | 0.728 | 0.621 | 0.67 | 0.673 | 0.054 |
| **Viperin** | 2016.Oax.02 | 6 | -6.88 | -5.27 | -6.08 | -6.077 | 0.805 |
| **Viperin** | 2016.Oax.02 | 12 | -8.103 | -5.577 | -6.84 | -6.84 | 1.263 |
| **Viperin** | 2016.Oax.02 | 24 | -6.524 | -5.624 | -6.07 | -6.072 | 0.45 |
| **Viperin** | 2016.Oax.02 | 48 | 2.949 | 2.39 | 2.67 | 2.67 | 0.279 |

## Supplementary Table S2. (Continued)

| Gene | Condition | Time (hpi) | Rep 1 log₂FC | Rep 2 log₂FC | Rep 3 log₂FC | Mean log₂FC | SD |
|---|---|---|---|---|---|---|---|
| **IFITM3** | NI | 24 | 0 | 0 | 0 | 0 | 0 |
| **IFITM3** | IFN-α A/D 10 IU | 6 | 1.5 | 1.72 | 1.61 | 1.61 | 0.11 |
| **IFITM3** | IFN-α A/D 10 IU | 12 | 4.32 | 4.26 | 4.29 | 4.29 | 0.03 |
| **IFITM3** | IFN-α A/D 10 IU | 24 | 2.93 | 3.01 | 2.97 | 2.97 | 0.04 |
| **IFITM3** | IFN-α A/D 10 IU | 48 | 6.11 | 5.92 | 6.01 | 6.01 | 0.1 |
| **IFITM3** | IFN-α A/D 100 IU | 6 | 4.6 | 4.72 | 4.66 | 4.66 | 0.06 |
| **IFITM3** | IFN-α A/D 100 IU | 12 | 3.14 | 3.09 | 3.21 | 3.14 | 0.06 |
| **IFITM3** | IFN-α A/D 100 IU | 24 | 3.85 | 3.57 | 4.14 | 3.85 | 0.29 |
| **IFITM3** | IFN-α A/D 100 IU | 48 | 10.78 | 10.82 | 11.05 | 10.88 | 0.15 |
| **IFITM3** | 2019.Oax.10 | 6 | 4.35 | 4.59 | 6.04 | 4.99 | 0.92 |
| **IFITM3** | 2019.Oax.10 | 12 | 3.73 | 4.06 | 3.44 | 3.74 | 0.31 |
| **IFITM3** | 2019.Oax.10 | 24 | 4.51 | 4.33 | 4.98 | 4.61 | 0.34 |
| **IFITM3** | 2019.Oax.10 | 48 | 2.71 | 2.62 | 2.73 | 2.69 | 0.06 |
| **IFITM3** | 2019.Oax.17 | 6 | 0.46 | 0.44 | 0.65 | 0.52 | 0.12 |
| **IFITM3** | 2019.Oax.17 | 12 | 3.6 | 3.6 | 3.75 | 3.65 | 0.09 |
| **IFITM3** | 2019.Oax.17 | 24 | 2.06 | 2.5 | 2.24 | 2.27 | 0.22 |
| **IFITM3** | 2019.Oax.17 | 48 | 3.29 | 2.75 | 2.48 | 2.84 | 0.41 |
| **IFITM3** | 2019.Oax.19 | 6 | -3.18 | -2.98 | -3.09 | -3.08 | 0.1 |
| **IFITM3** | 2019.Oax.19 | 12 | 2.66 | 2.94 | 3.08 | 2.89 | 0.21 |
| **IFITM3** | 2019.Oax.19 | 24 | 2.41 | 2.78 | 3.18 | 2.79 | 0.39 |
| **IFITM3** | 2019.Oax.19 | 48 | 1.44 | 1.7 | 3.67 | 2.27 | 1.23 |
| **IFITM3** | 2019.Oax.31 | 6 | -3.79 | -2.97 | -3.39 | -3.38 | 0.41 |
| **IFITM3** | 2019.Oax.31 | 12 | 0.48 | 0.82 | 0.5 | 0.6 | 0.19 |
| **IFITM3** | 2019.Oax.31 | 24 | 1.93 | 1.36 | 1.44 | 1.58 | 0.31 |
| **IFITM3** | 2019.Oax.31 | 48 | 2.45 | 1.27 | 2.39 | 2.04 | 0.66 |
| **IFITM3** | 2016.Oax.02 | 6 | -2.4 | -1.57 | -1.04 | -1.67 | 0.69 |
| **IFITM3** | 2016.Oax.02 | 12 | 0.74 | 1.74 | 0.66 | 1.05 | 0.6 |
| **IFITM3** | 2016.Oax.02 | 24 | 0.94 | 0.82 | 1.01 | 0.92 | 0.1 |
| **IFITM3** | 2016.Oax.02 | 48 | 3.65 | 3.78 | 3.21 | 3.55 | 0.3 |

## Supplementary Table S2. (Continued)

| Gene | Condition | Time (hpi) | Rep 1 $log_2FC$ | Rep 2 $log_2FC$ | Rep 3 $log_2FC$ | Mean $log_2FC$ | SD |
|---|---|---|---|---|---|---|---|
| **Mx1** | NI | 24 | 0 | 0 | 0 | 0 | 0 |
| **Mx1** | IFN-α A/D 10 IU | 6 | 9.52 | 9.53 | 9.47 | 9.51 | 0.03 |
| **Mx1** | IFN-α A/D 10 IU | 12 | 12.67 | 12.76 | 13.52 | 12.98 | 0.47 |
| **Mx1** | IFN-α A/D 10 IU | 24 | 6.04 | 5.98 | 6.01 | 6.01 | 0.03 |
| **Mx1** | IFN-α A/D 10 IU | 48 | 13.45 | 13.35 | 13.4 | 13.4 | 0.05 |
| **Mx1** | IFN-α A/D 100 IU | 6 | 2.14 | 1.87 | 1.97 | 1.99 | 0.14 |
| **Mx1** | IFN-α A/D 100 IU | 12 | 13.78 | 13.77 | 13.6 | 13.72 | 0.1 |
| **Mx1** | IFN-α A/D 100 IU | 24 | 1.6 | 1.72 | 1.66 | 1.66 | 0.06 |
| **Mx1** | IFN-α A/D 100 IU | 48 | 19.53 | 19.56 | 19.49 | 19.53 | 0.04 |
| **Mx1** | 2019.Oax.10 | 6 | 3.09 | 2.97 | 3.2 | 3.09 | 0.12 |
| **Mx1** | 2019.Oax.10 | 12 | 8.28 | 8.23 | 8.32 | 8.28 | 0.05 |
| **Mx1** | 2019.Oax.10 | 24 | 10.36 | 10.38 | 10.3 | 10.35 | 0.04 |
| **Mx1** | 2019.Oax.10 | 48 | 11.86 | 11.78 | 11.76 | 11.8 | 0.05 |
| **Mx1** | 2019.Oax.17 | 6 | 6.04 | 6.09 | 6.03 | 6.05 | 0.03 |
| **Mx1** | 2019.Oax.17 | 12 | 5.34 | 5.45 | 5.31 | 5.37 | 0.07 |
| **Mx1** | 2019.Oax.17 | 24 | 15.18 | 15.15 | 15.1 | 15.14 | 0.04 |
| **Mx1** | 2019.Oax.17 | 48 | 10.39 | 10.3 | 10.36 | 10.35 | 0.05 |
| **Mx1** | 2019.Oax.19 | 6 | 7.46 | 7.52 | 7.44 | 7.47 | 0.04 |
| **Mx1** | 2019.Oax.19 | 12 | 4.78 | 4.79 | 4.55 | 4.71 | 0.14 |
| **Mx1** | 2019.Oax.19 | 24 | 3.26 | 3.48 | 3.34 | 3.36 | 0.11 |
| **Mx1** | 2019.Oax.19 | 48 | 7.19 | 7.29 | 8.52 | 7.67 | 0.74 |
| **Mx1** | 2019.Oax.31 | 6 | 0.37 | 0.59 | 0.47 | 0.48 | 0.11 |
| **Mx1** | 2019.Oax.31 | 12 | 3.95 | 3.6 | 3.81 | 3.79 | 0.17 |
| **Mx1** | 2019.Oax.31 | 24 | 4.61 | 4.55 | 4.57 | 4.58 | 0.03 |
| **Mx1** | 2019.Oax.31 | 48 | 11.93 | 11.95 | 11.88 | 11.92 | 0.04 |
| **Mx1** | 2016.Oax.02 | 6 | -1.46 | -1.41 | -1.49 | -1.45 | 0.04 |
| **Mx1** | 2016.Oax.02 | 12 | 4.81 | 4.99 | 4.88 | 4.89 | 0.09 |
| **Mx1** | 2016.Oax.02 | 24 | 4.59 | 4.68 | 4.57 | 4.61 | 0.06 |
| **Mx1** | 2016.Oax.02 | 48 | 12.6 | 13.02 | 12.69 | 12.77 | 0.22 |

## Supplementary Table S2. (Continued)

| Gene | Condition | Time (hpi) | Rep 1 log₂FC | Rep 2 log₂FC | Rep 3 log₂FC | Mean log₂FC | SD |
|---|---|---|---|---|---|---|---|
| **IFNβ1** | NI | 24 | 0 | 0 | 0 | 0 | 0 |
| **IFNβ1** | IFN-α A/D 10 IU | 6 | -0.86 | -1 | -0.93 | -0.93 | 0.07 |
| **IFNβ1** | IFN-α A/D 10 IU | 12 | 4.72 | 4.59 | 4.66 | 4.66 | 0.07 |
| **IFNβ1** | IFN-α A/D 10 IU | 24 | 2.4 | 2.03 | 2.21 | 2.21 | 0.19 |
| **IFNβ1** | IFN-α A/D 10 IU | 48 | 2 | 2.03 | 2.02 | 2.02 | 0.02 |
| **IFNβ1** | IFN-α A/D 100 IU | 6 | -0.7 | -0.6 | -0.65 | -0.65 | 0.05 |
| **IFNβ1** | IFN-α A/D 100 IU | 12 | 1.77 | 2.06 | 1.91 | 1.91 | 0.14 |
| **IFNβ1** | IFN-α A/D 100 IU | 24 | 4.15 | 4.21 | 4.18 | 4.18 | 0.03 |
| **IFNβ1** | IFN-α A/D 100 IU | 48 | 6.38 | 6.2 | 6.29 | 6.29 | 0.09 |
| **IFNβ1** | 2019.Oax.10 | 6 | 3.21 | 3.07 | 3.14 | 3.14 | 0.07 |
| **IFNβ1** | 2019.Oax.10 | 12 | 0.59 | 0.41 | 0.5 | 0.5 | 0.09 |
| **IFNβ1** | 2019.Oax.10 | 24 | 3.51 | 3.61 | 3.56 | 3.56 | 0.05 |
| **IFNβ1** | 2019.Oax.10 | 48 | -2.15 | -2.18 | -2.17 | -2.17 | 0.02 |
| **IFNβ1** | 2019.Oax.17 | 6 | -2.05 | -2.82 | -2.44 | -2.44 | 0.39 |
| **IFNβ1** | 2019.Oax.17 | 12 | 4.13 | 4.3 | 4.21 | 4.21 | 0.09 |
| **IFNβ1** | 2019.Oax.17 | 24 | 3.53 | 3.5 | 3.52 | 3.52 | 0.02 |
| **IFNβ1** | 2019.Oax.17 | 48 | -5.31 | -3.99 | -4.65 | -4.65 | 0.66 |
| **IFNβ1** | 2019.Oax.19 | 6 | -6.62 | -6.56 | -6.59 | -6.59 | 0.03 |
| **IFNβ1** | 2019.Oax.19 | 12 | 0.03 | -0.32 | -0.14 | -0.14 | 0.18 |
| **IFNβ1** | 2019.Oax.19 | 24 | 4.8 | 4.73 | 4.77 | 4.77 | 0.04 |
| **IFNβ1** | 2019.Oax.19 | 48 | -7.71 | -2.86 | -5.29 | -5.29 | 2.43 |
| **IFNβ1** | 2019.Oax.31 | 6 | -6.67 | -6.34 | -6.5 | -6.5 | 0.17 |
| **IFNβ1** | 2019.Oax.31 | 12 | 0.05 | 0.9 | 0.48 | 0.48 | 0.43 |
| **IFNβ1** | 2019.Oax.31 | 24 | 2.67 | 2.8 | 2.73 | 2.73 | 0.07 |
| **IFNβ1** | 2019.Oax.31 | 48 | -0.29 | -4.15 | -2.22 | -2.22 | 1.93 |
| **IFNβ1** | 2016.Oax.02 | 6 | -3.18 | -2.91 | -3.05 | -3.05 | 0.14 |
| **IFNβ1** | 2016.Oax.02 | 12 | 0.63 | 0.26 | 0.45 | 0.45 | 0.18 |
| **IFNβ1** | 2016.Oax.02 | 24 | -0.21 | -0.01 | -0.11 | -0.11 | 0.1 |
| **IFNβ1** | 2016.Oax.02 | 48 | -5.15 | -3.29 | -4.22 | -4.22 | 0.93 |

**Supplementary Table S2. (Continued)**

| Gene | Condition | Time (hpi) | Rep 1 log₂FC | Rep 2 log₂FC | Rep 3 log₂FC | Mean log₂FC | SD |
|---|---|---|---|---|---|---|---|
| **IL8** | NI | 24 | 0 | 0 | 0 | 0 | 0 |
| **IL8** | IFN-α A/D 10 IU | 6 | -14.10 | -13.52 | -14.07 | -13.90 | 0.33 |
| **IL8** | IFN-α A/D 10 IU | 12 | 0.62 | 0.68 | 0.85 | 0.72 | 0.12 |
| **IL8** | IFN-α A/D 10 IU | 24 | -5.70 | -6.53 | -5.60 | -5.94 | 0.51 |
| **IL8** | IFN-α A/D 10 IU | 48 | 10.7 | 10.37 | 10.48 | 10.52 | 0.17 |
| **IL8** | IFN-α A/D 100 IU | 6 | -3.03 | -2.98 | -2.95 | -2.99 | 0.04 |
| **IL8** | IFN-α A/D 100 IU | 12 | 11.37 | 11.26 | 11.19 | 11.27 | 0.09 |
| **IL8** | IFN-α A/D 100 IU | 24 | -6.86 | -4.53 | -6.03 | -5.81 | 1.18 |
| **IL8** | IFN-α A/D 100 IU | 48 | 0.80 | 1 | 1 | 0.93 | 0.12 |
| **IL8** | 2019.Oax.10 | 6 | -3.45 | -3.77 | -3.59 | -3.6 | 0.16 |
| **IL8** | 2019.Oax.10 | 12 | 7.61 | 6.98 | 7.22 | 7.27 | 0.32 |
| **IL8** | 2019.Oax.10 | 24 | -6.66 | -6 | -6.45 | -6.37 | 0.34 |
| **IL8** | 2019.Oax.10 | 48 | -4.09 | -4.45 | -4.34 | -4.29 | 0.18 |
| **IL8** | 2019.Oax.17 | 6 | 0.10 | -0.26 | -0.28 | -0.15 | 0.21 |
| **IL8** | 2019.Oax.17 | 12 | 2.01 | 2.25 | 1.96 | 2.07 | 0.16 |
| **IL8** | 2019.Oax.17 | 24 | -7.35 | -8.21 | -7.67 | -7.74 | 0.43 |
| **IL8** | 2019.Oax.17 | 48 | -7.2 | -6.38 | -7 | -6.86 | 0.43 |
| **IL8** | 2019.Oax.19 | 6 | -3.23 | -2.88 | -2.95 | -3.02 | 0.19 |
| **IL8** | 2019.Oax.19 | 12 | -1.84 | -1.76 | -2.70 | -2.10 | 0.52 |
| **IL8** | 2019.Oax.19 | 24 | -3.21 | -4.22 | -3.64 | -3.69 | 0.51 |
| **IL8** | 2019.Oax.19 | 48 | -8.22 | -4.35 | -6.24 | -6.27 | 1.94 |
| **IL8** | 2019.Oax.31 | 6 | -3.39 | -4.14 | -3.87 | -3.8 | 0.38 |
| **IL8** | 2019.Oax.31 | 12 | -3.96 | -4.17 | -4.01 | -4.05 | 0.11 |
| **IL8** | 2019.Oax.31 | 24 | 6.86 | 6.77 | 6.72 | 6.78 | 0.07 |
| **IL8** | 2019.Oax.31 | 48 | -1.12 | -1.74 | -1.11 | -1.32 | 0.36 |
| **IL8** | 2016.Oax.02 | 6 | -4.06 | -5.65 | -5.55 | -5.09 | 0.89 |
| **IL8** | 2016.Oax.02 | 12 | -4.56 | -2.16 | -3.29 | -3.34 | 1.2 |
| **IL8** | 2016.Oax.02 | 24 | 2.21 | 1.80 | 2.03 | 2.01 | 0.21 |
| **IL8** | 2016.Oax.02 | 48 | 0.11 | 0.07 | 0.31 | 0.16 | 0.13 |
| **IL8** | LPS | 6 | 6.71 | 7.29 | 6.99 | 7 | 0.29 |

## Supplementary Table S2. (Continued)

| Gene | Condition | Time (hpi) | Rep 1 log₂FC | Rep 2 log₂FC | Rep 3 log₂FC | Mean log₂FC | SD |
|---|---|---|---|---|---|---|---|
| **TNFα** | NI | 24 | 0 | 0 | 0 | 0 | 0 |
| **TNFα** | IFN-α A/D 10 IU | 6 | -2.47 | -2.42 | -2.41 | -2.43 | 0.03 |
| **TNFα** | IFN-α A/D 10 IU | 12 | 8.47 | 8.29 | 8.44 | 8.40 | 0.1 |
| **TNFα** | IFN-α A/D 10 IU | 24 | -0.07 | -0.07 | 0.42 | 0.09 | 0.28 |
| **TNFα** | IFN-α A/D 10 IU | 48 | 15.15 | 14.95 | 15.04 | 15.05 | 0.1 |
| **TNFα** | IFN-α A/D 100 IU | 6 | -3.71 | -4.12 | -4 | -3.94 | 0.21 |
| **TNFα** | IFN-α A/D 100 IU | 12 | 16.09 | 16.29 | 16.16 | 16.18 | 0.1 |
| **TNFα** | IFN-α A/D 100 IU | 24 | -1.39 | -1.24 | -1.27 | -1.3 | 0.08 |
| **TNFα** | IFN-α A/D 100 IU | 48 | 11.98 | 12.12 | 11.99 | 12.03 | 0.08 |
| **TNFα** | 2019.Oax.10 | 6 | -4.16 | -4.05 | -4.14 | -4.12 | 0.06 |
| **TNFα** | 2019.Oax.10 | 12 | -3.17 | -3.92 | -3.7 | -3.6 | 0.39 |
| **TNFα** | 2019.Oax.10 | 24 | -0.72 | -0.79 | -0.79 | -0.77 | 0.04 |
| **TNFα** | 2019.Oax.10 | 48 | -4.94 | -4.07 | -4.63 | -4.55 | 0.44 |
| **TNFα** | 2019.Oax.17 | 6 | -6.43 | -5.88 | -6.07 | -6.13 | 0.28 |
| **TNFα** | 2019.Oax.17 | 12 | 13.43 | 13.58 | 13.45 | 13.49 | 0.08 |
| **TNFα** | 2019.Oax.17 | 24 | -3.17 | -2.50 | -2.92 | -2.86 | 0.34 |
| **TNFα** | 2019.Oax.17 | 48 | -11.16 | -10.10 | -10.42 | -10.56 | 0.54 |
| **TNFα** | 2019.Oax.19 | 6 | -10.06 | -8.97 | -9.52 | -9.52 | 0.55 |
| **TNFα** | 2019.Oax.19 | 12 | 11.23 | 11.31 | 11.04 | 11.19 | 0.14 |
| **TNFα** | 2019.Oax.19 | 24 | -0.95 | -0.34 | -0.64 | -0.64 | 0.31 |
| **TNFα** | 2019.Oax.19 | 48 | -0.76 | -1.07 | -1.11 | -0.98 | 0.19 |
| **TNFα** | 2019.Oax.31 | 6 | -9.18 | -8.19 | -8.71 | -8.69 | 0.5 |
| **TNFα** | 2019.Oax.31 | 12 | -1.60 | -1.15 | -1.33 | -1.36 | 0.23 |
| **TNFα** | 2019.Oax.31 | 24 | 3.56 | 3.20 | 3.33 | 3.36 | 0.18 |
| **TNFα** | 2019.Oax.31 | 48 | 5.59 | 5.62 | 5.48 | 5.56 | 0.07 |
| **TNFα** | 2016.Oax.02 | 6 | -3.48 | -1.92 | -2.75 | -2.72 | 0.78 |
| **TNFα** | 2016.Oax.02 | 12 | -3.02 | -2.05 | -2.68 | -2.58 | 0.49 |
| **TNFα** | 2016.Oax.02 | 24 | 9.83 | 10.43 | 10.05 | 10.10 | 0.31 |
| **TNFα** | 2016.Oax.02 | 48 | 7.90 | 9.19 | 8.15 | 8.41 | 0.68 |
| **TNFα** | LPS | 6 | 6.94 | 6.89 | 6.91 | 6.91 | 0.03 |

## Supplementary Table S2. (Continued)

| Gene | Condition | Time (hpi) | Rep 1 log₂FC | Rep 2 log₂FC | Rep 3 log₂FC | Mean log₂FC | SD |
|---|---|---|---|---|---|---|---|
| **RANTES** | NI | 24 | 0 | 0 | 0 | 0 | 0 |
| **RANTES** | IFN-α A/D 10 IU | 6 | -8.51 | -9.64 | -9.08 | -9.08 | 0.57 |
| **RANTES** | IFN-α A/D 10 IU | 12 | 6.87 | 6.90 | 6.89 | 6.89 | 0.02 |
| **RANTES** | IFN-α A/D 10 IU | 24 | -5.69 | -6.57 | -6.13 | -6.13 | 0.44 |
| **RANTES** | IFN-α A/D 10 IU | 48 | 12.66 | 12.63 | 12.64 | 12.64 | 0.02 |
| **RANTES** | IFN-α A/D 100 IU | 6 | -5.75 | -5.65 | -5.70 | -5.70 | 0.05 |
| **RANTES** | IFN-α A/D 100 IU | 12 | 8.78 | 8.94 | 8.86 | 8.86 | 0.08 |
| **RANTES** | IFN-α A/D 100 IU | 24 | -1.79 | -1.86 | -1.82 | -1.82 | 0.04 |
| **RANTES** | IFN-α A/D 100 IU | 48 | 4.59 | 4.51 | 4.55 | 4.55 | 0.04 |
| **RANTES** | 2019.Oax.10 | 6 | -1.88 | -1.87 | -1.87 | -1.87 | 0.01 |
| **RANTES** | 2019.Oax.10 | 12 | 7.04 | 7.10 | 7.07 | 7.07 | 0.03 |
| **RANTES** | 2019.Oax.10 | 24 | -2.68 | -3.50 | -3.09 | -3.09 | 0.41 |
| **RANTES** | 2019.Oax.10 | 48 | -9.46 | -9.16 | -9.31 | -9.31 | 0.15 |
| **RANTES** | 2019.Oax.17 | 6 | -5.79 | -5.99 | -5.89 | -5.89 | 0.1 |
| **RANTES** | 2019.Oax.17 | 12 | 14.44 | 14.48 | 14.46 | 14.46 | 0.02 |
| **RANTES** | 2019.Oax.17 | 24 | -5.62 | -4.70 | -5.16 | -5.16 | 0.46 |
| **RANTES** | 2019.Oax.17 | 48 | -8.26 | -9.17 | -8.71 | -8.71 | 0.46 |
| **RANTES** | 2019.Oax.19 | 6 | -11.38 | -11.82 | -11.6 | -11.6 | 0.22 |
| **RANTES** | 2019.Oax.19 | 12 | -4.81 | -5.78 | -5.29 | -5.29 | 0.48 |
| **RANTES** | 2019.Oax.19 | 24 | -5.3 | -5.26 | -5.28 | -5.28 | 0.02 |
| **RANTES** | 2019.Oax.19 | 48 | -9.45 | -9.72 | -9.59 | -9.59 | 0.14 |
| **RANTES** | 2019.Oax.31 | 6 | -11.83 | -12.40 | -12.11 | -12.11 | 0.29 |
| **RANTES** | 2019.Oax.31 | 12 | -5.99 | -5.98 | -5.98 | -5.98 | 0.01 |
| **RANTES** | 2019.Oax.31 | 24 | -1.43 | -2.19 | -1.81 | -1.81 | 0.38 |
| **RANTES** | 2016.Oax.02 | 6 | -8.62 | -9.63 | -9.13 | -9.13 | 0.51 |
| **RANTES** | 2016.Oax.02 | 12 | -3.90 | -3.30 | -3.60 | -3.60 | 0.3 |
| **RANTES** | 2016.Oax.02 | 24 | -2.51 | -2.06 | -2.29 | -2.29 | 0.23 |
| **RANTES** | 2016.Oax.02 | 48 | -8.50 | -7.87 | -8.19 | -8.19 | 0.32 |
| **RANTES** | LPS | 6 | -1.09 | -1.19 | -1.14 | -1.14 | 0.05 |

**Supplementary Table S2. (Continued)**

| Gene | Condition | Time (hpi) | Rep 1 $log_2FC$ | Rep 2 $log_2FC$ | Rep 3 $log_2FC$ | Mean $log_2FC$ | SD |
|---|---|---|---|---|---|---|---|
| **MCP-1** | NI | 24 | 0 | 0 | 0 | 0 | 0 |
| **MCP-1** | IFN-α A/D 10 IU | 6 | -1.66 | -2.02 | -1.84 | -1.84 | 0.18 |
| **MCP-1** | IFN-α A/D 10 IU | 12 | 0.62 | 0.19 | 0.41 | 0.41 | 0.22 |
| **MCP-1** | IFN-α A/D 10 IU | 24 | 1.73 | 3.33 | 2.53 | 2.53 | 0.8 |
| **MCP-1** | IFN-α A/D 10 IU | 48 | 2.2 | 2.2 | 2.2 | 2.2 | 0 |
| **MCP-1** | IFN-α A/D 100 IU | 6 | -1.52 | -0.98 | -1.25 | -1.25 | 0.27 |
| **MCP-1** | IFN-α A/D 100 IU | 12 | -0.93 | -0.61 | -0.77 | -0.77 | 0.16 |
| **MCP-1** | IFN-α A/D 100 IU | 24 | 2.05 | 1.97 | 2.01 | 2.01 | 0.04 |
| **MCP-1** | IFN-α A/D 100 IU | 48 | 7.48 | 7 | 7.24 | 7.24 | 0.24 |
| **MCP-1** | 2019.Oax.10 | 6 | 4.41 | 3.89 | 4.15 | 4.15 | 0.26 |
| **MCP-1** | 2019.Oax.10 | 12 | -1.37 | -2.12 | -1.75 | -1.75 | 0.38 |
| **MCP-1** | 2019.Oax.10 | 24 | 2.61 | 2.81 | 2.71 | 2.71 | 0.1 |
| **MCP-1** | 2019.Oax.10 | 48 | -1.99 | -1.87 | -1.93 | -1.93 | 0.06 |
| **MCP-1** | 2019.Oax.17 | 6 | 1.49 | 0.74 | 1.12 | 1.12 | 0.38 |
| **MCP-1** | 2019.Oax.17 | 12 | 1.23 | 1.5 | 1.36 | 1.36 | 0.14 |
| **MCP-1** | 2019.Oax.17 | 24 | 1.53 | 1.7 | 1.62 | 1.62 | 0.09 |
| **MCP-1** | 2019.Oax.17 | 48 | -2.16 | -1.05 | -1.60 | -1.60 | 0.56 |
| **MCP-1** | 2019.Oax.19 | 6 | -3.07 | -2.53 | -2.8 | -2.8 | 0.27 |
| **MCP-1** | 2019.Oax.19 | 12 | 0.23 | -1.76 | -0.76 | -0.76 | 1 |
| **MCP-1** | 2019.Oax.19 | 24 | 2.31 | 1.93 | 2.12 | 2.12 | 0.19 |
| **MCP-1** | 2019.Oax.19 | 48 | -4.26 | 0.34 | -1.96 | -1.96 | 2.3 |
| **MCP-1** | 2019.Oax.31 | 6 | -3.48 | -3.49 | -3.48 | -3.48 | 0.01 |
| **MCP-1** | 2019.Oax.31 | 12 | -1.80 | -1.79 | -1.79 | -1.79 | 0.01 |
| **MCP-1** | 2019.Oax.31 | 24 | -1.44 | -2 | -1.72 | -1.72 | 0.28 |
| **MCP-1** | 2019.Oax.31 | 48 | -2.12 | -4.28 | -3.2 | -3.2 | 1.08 |
| **MCP-1** | 2016.Oax.02 | 6 | -0.98 | -1.96 | -1.47 | -1.47 | 0.49 |
| **MCP-1** | 2016.Oax.02 | 12 | -2.17 | -0.12 | -1.15 | -1.15 | 1.03 |
| **MCP-1** | 2016.Oax.02 | 24 | -2.06 | -1.87 | -1.97 | -1.97 | 0.1 |
| **MCP-1** | 2016.Oax.02 | 48 | -3.05 | -1.93 | -2.49 | -2.49 | 0.56 |
| **MCP-1** | LPS | 6 | 18.90 | 18.90 | 18.90 | 18.90 | 0 |

Notes: Relative gene expression in HaCaT cells was determined by RT-qPCR after infection with the indicated viral isolates or treatment with IFN-α A/D or LPS, as applicable. Expression values were calculated using the comparative $2^{-\Delta\Delta Ct}$ method, with GAPDH as the endogenous reference gene. Relative expression values at each post-infection time point were normalized to the corresponding time-matched non-infected (NI) control processed in parallel, which served as the calibrator. Values are presented as $log_2$ fold change. Data are shown as mean ± SD of three independent biological replicates unless otherwise indicated. For the RT-qPCR positive-control

experiments, IFN-α A/D was applied at 10 or 100 IU/mL for 6, 12, 24, or 48 h, whereas LPS was applied at 1 µg/mL for 6 h. These treatment groups served as independent positive controls for antiviral and inflammatory transcriptional responses, respectively, and were not included in the integrated heatmap analysis because they were not experimental comparison groups.
For simplicity, only one representative non-infected (NI) condition is shown.

**Supplementary Table S3. Two-way ANOVA and Tukey's multiple-comparison analysis of RT-qPCR gene expression in HaCaT cells infected with the indicated viral isolates.**

| Gene | Time (hpi) | Comparison | Mean difference | Adjusted p-value | Significance |
|---|---|---|---|---|---|
| **OAS1** | 6 | 2019.Oax.10 vs. 2019.Oax.17 | -3.69 | 0.0005 | *** |
| **OAS1** | 6 | 2019.Oax.10 vs. 2019.Oax.19 | 0.3567 | 0.9809 | ns |
| **OAS1** | 6 | 2019.Oax.10 vs. 2019.Oax.31 | 0.24 | 0.6993 | ns |
| **OAS1** | 6 | 2019.Oax.10 vs. 2016.Oax.02 | 2.307 | 0.0308 | * |
| **OAS1** | 6 | 2019.Oax.17 vs. 2019.Oax.19 | 4.047 | 0.0769 | ns |
| **OAS1** | 6 | 2019.Oax.17 vs. 2019.Oax.31 | 3.93 | 0.0036 | ** |
| **OAS1** | 6 | 2019.Oax.17 vs. 2016.Oax.02 | 5.997 | 0.0027 | ** |
| **OAS1** | 6 | 2019.Oax.19 vs. 2019.Oax.31 | -0.1167 | 0.9997 | ns |
| **OAS1** | 6 | 2019.Oax.19 vs. 2016.Oax.02 | 1.95 | 0.2944 | ns |
| **OAS1** | 6 | 2019.Oax.31 vs. 2016.Oax.02 | 2.067 | 0.0716 | ns |
| **OAS1** | 12 | 2019.Oax.10 vs. 2019.Oax.17 | -1.223 | 0.0021 | ** |
| **OAS1** | 12 | 2019.Oax.10 vs. 2019.Oax.19 | 1.78 | 0.0046 | ** |
| **OAS1** | 12 | 2019.Oax.10 vs. 2019.Oax.31 | 2.713 | 0.0001 | *** |
| **OAS1** | 12 | 2019.Oax.10 vs. 2016.Oax.020 | 1.613 | 0.0834 | ns |
| **OAS1** | 12 | 2019.Oax.17 vs. 2019.Oax.19 | 3.003 | <0.0001 | **** |
| **OAS1** | 12 | 2019.Oax.17 vs. 2019.Oax.31 | 3.937 | <0.0001 | **** |
| **OAS1** | 12 | 2019.Oax.17 vs. 2016.Oax.02 | 2.837 | 0.0264 | * |
| **OAS1** | 12 | 2019.Oax.19 vs. 2019.Oax.31 | 0.9333 | 0.0171 | * |
| **OAS1** | 12 | 2019.Oax.19 vs. 2016.Oax.02 | -0.1667 | 0.9709 | ns |
| **OAS1** | 12 | 2019.Oax.31 vs. 2016.Oax.02 | -1.1 | 0.1806 | ns |
| **OAS1** | 24 | 2019.Oax.10 vs. 2019.Oax.17 | 0.5467 | 0.8062 | ns |
| **OAS1** | 24 | 2019.Oax.10 vs. 2019.Oax.19 | 1.12 | 0.0009 | *** |
| **OAS1** | 24 | 2019.Oax.10 vs. 2019.Oax.31 | 0.0667 | 0.9816 | ns |
| **OAS1** | 24 | 2019.Oax.10 vs. 2016.Oax.02 | 0.9467 | 0.0083 | ** |
| **OAS1** | 24 | 2019.Oax.17 vs. 2019.Oax.19 | 0.5733 | 0.7855 | ns |
| **OAS1** | 24 | 2019.Oax.17 vs. 2019.Oax.31 | -0.48 | 0.8658 | ns |
| **OAS1** | 24 | 2019.Oax.17 vs. 2016.Oax.020 | 0.4 | 0.9102 | ns |
| **OAS1** | 24 | 2019.Oax.19 vs. 2019.Oax.31 | -1.053 | 0.0185 | * |
| **OAS1** | 24 | 2019.Oax.19 vs. 2016.Oax.020 | -0.1733 | 0.2908 | ns |

| | | | | | |
|---|---|---|---|---|---|
| **OAS1** | 24 | 2019.Oax.31 vs. 2016.Oax.02 | 0.88 | 0.0547 | ns |
| **OAS1** | 48 | 2019.Oax.10 vs. 2019.Oax.17 | 0.6333 | 0.0112 | * |
| **OAS1** | 48 | 2019.Oax.10 vs. 2019.Oax.19 | 2.153 | <0.0001 | **** |
| **OAS1** | 48 | 2019.Oax.10 vs. 2019.Oax.31 | 1.877 | 0.0002 | *** |
| **OAS1** | 48 | 2019.Oax.10 vs. 2016.Oax.02 | 1.797 | 0.0003 | *** |

## Supplementary Table S3. (Continued)

| Gene | Time (hpi) | Comparison | Mean difference | Adjusted p-value | Significance |
|---|---|---|---|---|---|
| **OAS1** | 48 | 2019.Oax.10 vs. 2016.Oax.02 | 1.797 | 0.0003 | *** |
| **OAS1** | 48 | 2019.Oax.17 vs. 2019.Oax.19 | 1.52 | <0.0001 | **** |
| **OAS1** | 48 | 2019.Oax.17 vs. 2019.Oax.31 | 1.243 | 0.0058 | ** |
| **OAS1** | 48 | 2019.Oax.17 vs. 2016.Oax.02 | 1.163 | 0.0075 | ** |
| **OAS1** | 48 | 2019.Oax.19 vs. 2019.Oax.31 | -0.2767 | 0.1377 | ns |
| **OAS1** | 48 | 2019.Oax.19 vs. 2016.Oax.02 | -0.3567 | 0.0939 | ns |
| **OAS1** | 48 | 2019.Oax.31 vs. 2016.Oax.020 | -0.08 | 0.8959 | ns |
| **PKR** | 6 | 2019.Oax.10 vs 2019.Oax.17 | 2.58 | 0.1034 | ns |
| **PKR** | 6 | 2019.Oax.10 vs 2019.Oax.19 | 3.73 | 0.0262 | * |
| **PKR** | 6 | 2019.Oax.10 vs 2019.Oax.31 | 3.15 | 0.0646 | ns |
| **PKR** | 6 | 2019.Oax.10 vs 2016.Oax.02 | 4.317 | 0.0279 | * |
| **PKR** | 6 | 2019.Oax.17 vs 2019.Oax.19 | 1.15 | 0.4535 | ns |
| **PKR** | 6 | 2019.Oax.17 vs 2019.Oax.31 | 0.57 | 0.9081 | ns |
| **PKR** | 6 | 2019.Oax.17 vs 2016.Oax.02 | 1.737 | 0.2227 | ns |
| **PKR** | 6 | 2019.Oax.19 vs 2019.Oax.31 | -0.58 | 0.8533 | ns |
| **PKR** | 6 | 2019.Oax.19 vs 2016.Oax.02 | 0.587 | 0.8200 | ns |
| **PKR** | 6 | 2019.Oax.31 vs 2016.Oax.02 | 1.167 | 0.4637 | ns |
| **PKR** | 12 | 2019.Oax.10 vs 2019.Oax.17 | -0.156 | 0.2256 | ns |
| **PKR** | 12 | 2019.Oax.10 vs 2019.Oax.19 | 4.735 | 0.0041 | ** |
| **PKR** | 12 | 2019.Oax.10 vs 2019.Oax.31 | 4.742 | 0.0026 | ** |
| **PKR** | 12 | 2019.Oax.10 vs 2016.Oax.02 | 3.916 | <0.0001 | **** |
| **PKR** | 12 | 2019.Oax.17 vs 2019.Oax.19 | 4.891 | 0.0048 | ** |
| **PKR** | 12 | 2019.Oax.17 vs 2019.Oax.31 | 4.898 | 0.0031 | ** |
| **PKR** | 12 | 2019.Oax.17 vs 2016.Oax.02 | 4.072 | <0.0001 | **** |
| **PKR** | 12 | 2019.Oax.19 vs 2019.Oax.31 | 0.007 | >0.9999 | ns |
| **PKR** | 12 | 2019.Oax.19 vs 2016.Oax.02 | -0.818 | 0.1662 | ns |
| **PKR** | 12 | 2019.Oax.31 vs 2016.Oax.02 | -0.826 | 0.1314 | ns |
| **PKR** | 24 | 2019.Oax.10 vs 2019.Oax.17 | 3.05 | 0.0982 | ns |
| **PKR** | 24 | 2019.Oax.10 vs 2019.Oax.19 | 0.107 | 0.9670 | ns |
| **PKR** | 24 | 2019.Oax.10 vs 2019.Oax.31 | 1.21 | 0.1554 | ns |

| | | | | | |
|---|---|---|---|---|---|
| **PKR** | 24 | 2019.Oax.10 vs 2016.Oax.02 | 0.942 | 0.0447 | * |
| **PKR** | 24 | 2019.Oax.17 vs 2019.Oax.19 | -2.943 | 0.0955 | ns |
| **PKR** | 24 | 2019.Oax.17 vs 2019.Oax.31 | -1.84 | 0.2310 | ns |
| **PKR** | 24 | 2019.Oax.17 vs 2016.Oax.02 | -2.108 | 0.1856 | ns |

## Supplementary Table S3. (Continued)

| Gene | Time (hpi) | Comparison | Mean difference | Adjusted p-value | Significance |
|---|---|---|---|---|---|
| **PKR** | 24 | 2019.Oax.19 vs 2019.Oax.31 | 1.104 | 0.1727 | ns |
| **PKR** | 24 | 2019.Oax.19 vs 2016.Oax.02 | 0.836 | 0.0830 | ns |
| **PKR** | 24 | 2019.Oax.31 vs 2016.Oax.02 | -0.268 | 0.9191 | ns |
| **PKR** | 48 | 2019.Oax.10 vs 2019.Oax.17 | 0.813 | 0.0028 | ** |
| **PKR** | 48 | 2019.Oax.10 vs 2019.Oax.19 | 1.967 | 0.1024 | ns |
| **PKR** | 48 | 2019.Oax.10 vs 2019.Oax.31 | -0.08 | 0.9917 | ns |
| **PKR** | 48 | 2019.Oax.10 vs 2016.Oax.02 | -0.26 | 0.1563 | ns |
| **PKR** | 48 | 2019.Oax.17 vs 2019.Oax.19 | 1.153 | 0.2666 | ns |
| **PKR** | 48 | 2019.Oax.17 vs 2019.Oax.31 | -0.893 | 0.1153 | ns |
| **PKR** | 48 | 2019.Oax.17 vs 2016.Oax.02 | -1.073 | 0.0009 | *** |
| **PKR** | 48 | 2019.Oax.19 vs 2019.Oax.31 | -2.046 | 0.0684 | ns |
| **PKR** | 48 | 2019.Oax.19 vs 2016.Oax.02 | -2.227 | 0.0806 | ns |
| **PKR** | 48 | 2019.Oax.31 vs 2016.Oax.02 | -0.18 | 0.8816 | ns |
| **Viperin** | 6 | 2019.Oax.10 vs. 2019.Oax.17 | 6.03 | <0.0001 | **** |
| **Viperin** | 6 | 2019.Oax.10 vs. 2019.Oax.19 | 7.603 | <0.0001 | **** |
| **Viperin** | 6 | 2019.Oax.10 vs. 2019.Oax.31 | 6.13 | 0.0216 | * |
| **Viperin** | 6 | 2019.Oax.10 vs. 2016.Oax.02 | 5.287 | 0.0160 | * |
| **Viperin** | 6 | 2019.Oax.17 vs. 2019.Oax.19 | 1.573 | 0.0056 | ** |
| **Viperin** | 6 | 2019.Oax.17 vs. 2019.Oax.31 | 0.1 | 0.9996 | ns |
| **Viperin** | 6 | 2019.Oax.17 vs. 2016.Oax.02 | -0.743 | 0.6100 | ns |
| **Viperin** | 6 | 2019.Oax.19 vs. 2019.Oax.31 | -1.473 | 0.3492 | ns |
| **Viperin** | 6 | 2019.Oax.19 vs. 2016.Oax.02 | -2.317 | 0.1130 | ns |
| **Viperin** | 6 | 2019.Oax.31 vs. 2016.Oax.02 | -0.843 | 0.7869 | ns |
| **Viperin** | 12 | 2019.Oax.10 vs. 2019.Oax.17 | 8.401 | 0.0043 | ** |
| **Viperin** | 12 | 2019.Oax.10 vs. 2019.Oax.19 | 11.56 | 0.0002 | *** |
| **Viperin** | 12 | 2019.Oax.10 vs. 2019.Oax.31 | 13.97 | 0.0053 | ** |
| **Viperin** | 12 | 2019.Oax.10 vs. 2016.Oax.02 | 12.61 | 0.0107 | * |
| **Viperin** | 12 | 2019.Oax.17 vs. 2019.Oax.19 | 3.157 | 0.0087 | ** |
| **Viperin** | 12 | 2019.Oax.17 vs. 2019.Oax.31 | 5.571 | 0.0119 | * |
| **Viperin** | 12 | 2019.Oax.17 vs. 2016.Oax.02 | 4.209 | 0.0595 | ns |

| | | | | | |
|---|---|---|---|---|---|
| **Viperin** | 12 | 2019.Oax.19 vs. 2019.Oax.31 | 2.414 | 0.1257 | ns |
| **Viperin** | 12 | 2019.Oax.19 vs. 2016.Oax.02 | 1.052 | 0.6933 | ns |
| **Viperin** | 12 | 2019.Oax.31 vs. 2016.Oax.02 | -1.362 | 0.6287 | ns |
| **Viperin** | 24 | 2019.Oax.10 vs. 2019.Oax.17 | 1.64 | 0.2230 | ns |

## Supplementary Table S3. (Continued)

| Gene | Time (hpi) | Comparison | Mean difference | Adjusted p-value | Significance |
|---|---|---|---|---|---|
| **Viperin** | 24 | 2019.Oax.10 vs. 2019.Oax.19 | 0.06 | 0.9997 | ns |
| **Viperin** | 24 | 2019.Oax.10 vs. 2019.Oax.31 | 0.55 | 0.4559 | ns |
| **Viperin** | 24 | 2019.Oax.10 vs. 2016.Oax.02 | 1.232 | 0.0941 | ns |
| **Viperin** | 24 | 2019.Oax.17 vs. 2019.Oax.19 | -1.58 | 0.2408 | ns |
| **Viperin** | 24 | 2019.Oax.17 vs. 2019.Oax.31 | -1.09 | 0.4484 | ns |
| **Viperin** | 24 | 2019.Oax.17 vs. 2016.Oax.02 | -0.408 | 0.9398 | ns |
| **Viperin** | 24 | 2019.Oax.19 vs. 2019.Oax.31 | 0.49 | 0.6488 | ns |
| **Viperin** | 24 | 2019.Oax.19 vs. 2016.Oax.02 | 1.172 | 0.1456 | ns |
| **Viperin** | 24 | 2019.Oax.31 vs. 2016.Oax.02 | 0.682 | 0.3924 | ns |
| **Viperin** | 48 | 2019.Oax.10 vs. 2019.Oax.17 | 1.01 | 0.0024 | ** |
| **Viperin** | 48 | 2019.Oax.10 vs. 2019.Oax.19 | 5.52 | 0.0033 | ** |
| **Viperin** | 48 | 2019.Oax.10 vs. 2019.Oax.31 | 4.347 | <0.0001 | **** |
| **Viperin** | 48 | 2019.Oax.10 vs. 2016.Oax.020 | 2.35 | 0.0144 | * |
| **Viperin** | 48 | 2019.Oax.17 vs. 2019.Oax.19 | 4.51 | 0.0050 | ** |
| **Viperin** | 48 | 2019.Oax.17 vs. 2019.Oax.31 | 3.337 | <0.0001 | **** |
| **Viperin** | 48 | 2019.Oax.17 vs. 2016.Oax.02 | 1.34 | 0.0365 | * |
| **Viperin** | 48 | 2019.Oax.19 vs. 2019.Oax.31 | -1.173 | 0.0759 | ns |
| **Viperin** | 48 | 2019.Oax.19 vs. 2016.Oax.02 | -3.17 | 0.0014 | ** |
| **Viperin** | 48 | 2019.Oax.31 vs. 2016.Oax.02 | -1.997 | 0.0164 | * |
| **IFITM3** | 6 | 2019.Oax.10 vs. 2019.Oax.17 | 4.477 | 0.0390 | * |
| **IFITM3** | 6 | 2019.Oax.10 vs. 2019.Oax.19 | 8.077 | 0.0123 | * |
| **IFITM3** | 6 | 2019.Oax.10 vs. 2019.Oax.31 | 8.377 | 0.0041 | ** |
| **IFITM3** | 6 | 2019.Oax.10 vs. 2016.Oax.02 | 6.663 | 0.0036 | ** |
| **IFITM3** | 6 | 2019.Oax.17 vs. 2019.Oax.19 | 3.6 | <0.0001 | **** |
| **IFITM3** | 6 | 2019.Oax.17 vs. 2019.Oax.31 | 3.9 | 0.0071 | ** |
| **IFITM3** | 6 | 2019.Oax.17 vs. 2016.Oax.02 | 2.187 | 0.0878 | ns |
| **IFITM3** | 6 | 2019.Oax.19 vs. 2019.Oax.31 | 0.3 | 0.7475 | ns |
| **IFITM3** | 6 | 2019.Oax.19 vs. 2016.Oax.02 | -1.413 | 0.1992 | ns |
| **IFITM3** | 6 | 2019.Oax.31 vs. 2016.Oax.02 | -1.713 | 0.1123 | ns |
| **IFITM3** | 12 | 2019.Oax.10 vs. 2019.Oax.17 | 0.094 | 0.9797 | ns |

| | | | | | |
|---|---|---|---|---|---|
| **IFITM3** | 12 | 2019.Oax.10 vs. 2019.Oax.19 | 0.848 | 0.0895 | ns |
| **IFITM3** | 12 | 2019.Oax.10 vs. 2019.Oax.31 | 3.142 | 0.0015 | ** |
| **IFITM3** | 12 | 2019.Oax.10 vs. 2016.Oax.02 | 2.696 | 0.0249 | * |
| **IFITM3** | 12 | 2019.Oax.17 vs. 2019.Oax.19 | 0.754 | 0.0539 | ns |

## Supplementary Table S3. (Continued)

| Gene | Time (hpi) | Comparison | Mean difference | Adjusted p-value | Significance |
|---|---|---|---|---|---|
| **IFITM3** | 12 | 2019.Oax.17 vs. 2019.Oax.31 | 3.048 | 0.0011 | ** |
| **IFITM3** | 12 | 2019.Oax.17 vs. 2016.Oax.02 | 2.602 | 0.0496 | * |
| **IFITM3** | 12 | 2019.Oax.19 vs. 2019.Oax.31 | 2.294 | 0.0008 | *** |
| **IFITM3** | 12 | 2019.Oax.19 vs. 2016.Oax.02 | 1.848 | 0.0805 | ns |
| **IFITM3** | 12 | 2019.Oax.31 vs. 2016.Oax.02 | -0.446 | 0.7529 | ns |
| **IFITM3** | 24 | 2019.Oax.10 vs. 2019.Oax.17 | 2.34 | 0.0047 | ** |
| **IFITM3** | 24 | 2019.Oax.10 vs. 2019.Oax.19 | 1.817 | 0.0171 | * |
| **IFITM3** | 24 | 2019.Oax.10 vs. 2019.Oax.31 | 3.03 | 0.0016 | ** |
| **IFITM3** | 24 | 2019.Oax.10 vs. 2016.Oax.02 | 3.686 | 0.0051 | ** |
| **IFITM3** | 24 | 2019.Oax.17 vs. 2019.Oax.19 | -0.523 | 0.4090 | ns |
| **IFITM3** | 24 | 2019.Oax.17 vs. 2019.Oax.31 | 0.69 | 0.1565 | ns |
| **IFITM3** | 24 | 2019.Oax.17 vs. 2016.Oax.02 | 1.346 | 0.0125 | * |
| **IFITM3** | 24 | 2019.Oax.19 vs. 2019.Oax.31 | 1.214 | 0.0620 | ns |
| **IFITM3** | 24 | 2019.Oax.19 vs. 2016.Oax.02 | 1.869 | 0.0334 | * |
| **IFITM3** | 24 | 2019.Oax.31 vs. 2016.Oax.02 | 0.655 | 0.1796 | ns |
| **IFITM3** | 48 | 2019.Oax.10 vs. 2019.Oax.17 | -0.153 | 0.9548 | ns |
| **IFITM3** | 48 | 2019.Oax.10 vs. 2019.Oax.19 | 0.417 | 0.9642 | ns |
| **IFITM3** | 48 | 2019.Oax.10 vs. 2019.Oax.31 | 0.65 | 0.5728 | ns |
| **IFITM3** | 48 | 2019.Oax.10 vs. 2016.Oax.02 | -0.86 | 0.1052 | ns |
| **IFITM3** | 48 | 2019.Oax.17 vs. 2019.Oax.19 | 0.57 | 0.9231 | ns |
| **IFITM3** | 48 | 2019.Oax.17 vs. 2019.Oax.31 | 0.804 | 0.4982 | ns |
| **IFITM3** | 48 | 2019.Oax.17 vs. 2016.Oax.02 | -0.707 | 0.2888 | ns |
| **IFITM3** | 48 | 2019.Oax.19 vs. 2019.Oax.31 | 0.234 | 0.9975 | ns |
| **IFITM3** | 48 | 2019.Oax.19 vs. 2016.Oax.02 | -1.277 | 0.5397 | ns |
| **IFITM3** | 48 | 2019.Oax.31 vs. 2016.Oax.02 | -1.51 | 0.1455 | ns |
| **Mx1** | 6 | 2019.Oax.10 vs. 2019.Oax.17 | -2.967 | <0.0001 | **** |
| **Mx1** | 6 | 2019.Oax.10 vs. 2019.Oax.19 | -4.387 | <0.0001 | **** |
| **Mx1** | 6 | 2019.Oax.10 vs. 2019.Oax.31 | 2.61 | <0.0001 | **** |
| **Mx1** | 6 | 2019.Oax.10 vs. 2016.Oax.02 | 4.54 | <0.0001 | **** |
| **Mx1** | 6 | 2019.Oax.17 vs. 2019.Oax.19 | -1.42 | <0.0001 | **** |

| | | | | | |
|---|---|---|---|---|---|
| **Mx1** | 6 | 2019.Oax.17 vs. 2019.Oax.31 | 5.577 | <0.0001 | **** |
| **Mx1** | 6 | 2019.Oax.17 vs. 2016.Oax.02 | 7.507 | <0.0001 | **** |
| **Mx1** | 6 | 2019.Oax.19 vs. 2019.Oax.31 | 6.997 | <0.0001 | **** |
| **Mx1** | 6 | 2019.Oax.19 vs. 2016.Oax.02 | 8.927 | <0.0001 | **** |

## Supplementary Table S3. (Continued)

| Gene | Time (hpi) | Comparison | Mean difference | Adjusted p-value | Significance |
|---|---|---|---|---|---|
| **Mx1** | 6 | 2019.Oax.31 vs. 2016.Oax.02 | 1.93 | 0.0010 | ** |
| **Mx1** | 12 | 2019.Oax.10 vs. 2019.Oax.17 | 2.909 | <0.0001 | **** |
| **Mx1** | 12 | 2019.Oax.10 vs. 2019.Oax.19 | 3.568 | <0.0001 | **** |
| **Mx1** | 12 | 2019.Oax.10 vs. 2019.Oax.31 | 4.489 | <0.0001 | **** |
| **Mx1** | 12 | 2019.Oax.10 vs. 2016.Oax.02 | 3.382 | <0.0001 | **** |
| **Mx1** | 12 | 2019.Oax.17 vs. 2019.Oax.19 | 0.6591 | 0.0194 | * |
| **Mx1** | 12 | 2019.Oax.17 vs. 2019.Oax.31 | 1.58 | 0.0047 | ** |
| **Mx1** | 12 | 2019.Oax.17 vs. 2016.Oax.02 | 0.4736 | 0.0116 | * |
| **Mx1** | 12 | 2019.Oax.19 vs. 2019.Oax.31 | 0.9211 | 0.0110 | * |
| **Mx1** | 12 | 2019.Oax.19 vs. 2016.Oax.02 | -0.1854 | 0.4312 | ns |
| **Mx1** | 12 | 2019.Oax.31 vs. 2016.Oax.02 | -1.107 | 0.0089 | ** |
| **Mx1** | 24 | 2019.Oax.10 vs. 2019.Oax.17 | -4.797 | <0.0001 | **** |
| **Mx1** | 24 | 2019.Oax.10 vs. 2019.Oax.19 | 6.987 | <0.0001 | **** |
| **Mx1** | 24 | 2019.Oax.10 vs. 2019.Oax.31 | 5.77 | <0.0001 | **** |
| **Mx1** | 24 | 2019.Oax.10 vs. 2016.Oax.02 | 5.733 | <0.0001 | **** |
| **Mx1** | 24 | 2019.Oax.17 vs. 2019.Oax.19 | 11.78 | <0.0001 | **** |
| **Mx1** | 24 | 2019.Oax.17 vs. 2019.Oax.31 | 10.57 | <0.0001 | **** |
| **Mx1** | 24 | 2019.Oax.17 vs. 2016.Oax.02 | 10.53 | <0.0001 | **** |
| **Mx1** | 24 | 2019.Oax.19 vs. 2019.Oax.31 | -1.216 | 0.0052 | ** |
| **Mx1** | 24 | 2019.Oax.19 vs. 2016.Oax.02 | -1.253 | 0.0019 | ** |
| **Mx1** | 24 | 2019.Oax.31 vs. 2016.Oax.02 | -0.03723 | 0.8436 | ns |
| **Mx1** | 48 | 2019.Oax.10 vs. 2019.Oax.17 | 1.45 | <0.0001 | **** |
| **Mx1** | 48 | 2019.Oax.10 vs. 2019.Oax.19 | 4.133 | 0.0314 | * |
| **Mx1** | 48 | 2019.Oax.10 vs. 2019.Oax.31 | -0.1194 | 0.1482 | ns |
| **Mx1** | 48 | 2019.Oax.10 vs. 2016.Oax.02 | -0.97 | 0.0428 | * |
| **Mx1** | 48 | 2019.Oax.17 vs. 2019.Oax.19 | 2.683 | 0.0730 | ns |
| **Mx1** | 48 | 2019.Oax.17 vs. 2019.Oax.31 | -1.569 | <0.0001 | **** |
| **Mx1** | 48 | 2019.Oax.17 vs. 2016.Oax.02 | -2.42 | 0.0068 | ** |
| **Mx1** | 48 | 2019.Oax.19 vs. 2019.Oax.31 | -4.253 | 0.0301 | * |
| **Mx1** | 48 | 2019.Oax.19 vs. 2016.Oax.02 | -5.103 | 0.0138 | * |

| | | | | | |
|---|---|---|---|---|---|
| **Mx1** | 48 | 2019.Oax.31 vs. 2016.Oax.02 | -0.8506 | 0.0614 | ns |
| **IFNβ1** | 6 | 2019.Oax.10 vs. 2019.Oax.17 | 5.577 | 0.0039 | ** |
| **IFNβ1** | 6 | 2019.Oax.10 vs. 2019.Oax.19 | 9.73 | <0.0001 | **** |
| **IFNβ1** | 6 | 2019.Oax.10 vs. 2019.Oax.31 | 9.643 | <0.0001 | **** |

**Supplementary Table S3. (Continued)**

| Gene | Time (hpi) | Comparison | Mean difference | Adjusted p-value | Significance |
|---|---|---|---|---|---|
| **IFNβ1** | 6 | 2019.Oax.10 vs. 2016.Oax.02 | 6.187 | <0.0001 | **** |
| **IFNβ1** | 6 | 2019.Oax.17 vs. 2019.Oax.19 | 4.153 | 0.0089 | ** |
| **IFNβ1** | 6 | 2019.Oax.17 vs. 2019.Oax.31 | 4.067 | 0.0031 | ** |
| **IFNβ1** | 6 | 2019.Oax.17 vs. 2016.Oax.02 | 0.61 | 0.3016 | ns |
| **IFNβ1** | 6 | 2019.Oax.19 vs. 2019.Oax.31 | -0.08667 | 0.8799 | ns |
| **IFNβ1** | 6 | 2019.Oax.19 vs. 2016.Oax.02 | -3.543 | <0.0001 | **** |
| **IFNβ1** | 6 | 2019.Oax.31 vs. 2016.Oax.02 | -3.457 | <0.0001 | **** |
| **IFNβ1** | 12 | 2019.Oax.10 vs. 2019.Oax.17 | -3.713 | <0.0001 | **** |
| **IFNβ1** | 12 | 2019.Oax.10 vs. 2019.Oax.19 | 0.6412 | 0.0421 | * |
| **IFNβ1** | 12 | 2019.Oax.10 vs. 2019.Oax.31 | 0.02167 | >0.9999 | ns |
| **IFNβ1** | 12 | 2019.Oax.10 vs. 2016.Oax.02 | 0.05287 | 0.987 | ns |
| **IFNβ1** | 12 | 2019.Oax.17 vs. 2019.Oax.19 | 4.354 | <0.0001 | **** |
| **IFNβ1** | 12 | 2019.Oax.17 vs. 2019.Oax.31 | 3.734 | 0.0106 | * |
| **IFNβ1** | 12 | 2019.Oax.17 vs. 2016.Oax.02 | 3.766 | 0.0003 | *** |
| **IFNβ1** | 12 | 2019.Oax.19 vs. 2019.Oax.31 | -0.6195 | 0.3516 | ns |
| **IFNβ1** | 12 | 2019.Oax.19 vs. 2016.Oax.02 | -0.5883 | 0.0674 | ns |
| **IFNβ1** | 12 | 2019.Oax.31 vs. 2016.Oax.02 | 0.0312 | >0.9999 | ns |
| **IFNβ1** | 24 | 2019.Oax.10 vs. 2019.Oax.17 | 0.04333 | 0.6605 | ns |
| **IFNβ1** | 24 | 2019.Oax.10 vs. 2019.Oax.19 | -1.207 | <0.0001 | **** |
| **IFNβ1** | 24 | 2019.Oax.10 vs. 2019.Oax.31 | 0.8271 | 0.0004 | *** |
| **IFNβ1** | 24 | 2019.Oax.10 vs. 2016.Oax.02 | 3.672 | <0.0001 | **** |
| **IFNβ1** | 24 | 2019.Oax.17 vs. 2019.Oax.19 | -1.25 | <0.0001 | **** |
| **IFNβ1** | 24 | 2019.Oax.17 vs. 2019.Oax.31 | 0.7838 | 0.0047 | ** |
| **IFNβ1** | 24 | 2019.Oax.17 vs. 2016.Oax.02 | 3.629 | <0.0001 | **** |
| **IFNβ1** | 24 | 2019.Oax.19 vs. 2019.Oax.31 | 2.034 | <0.0001 | **** |
| **IFNβ1** | 24 | 2019.Oax.19 vs. 2016.Oax.02 | 4.879 | <0.0001 | **** |
| **IFNβ1** | 24 | 2019.Oax.31 vs. 2016.Oax.02 | 2.845 | <0.0001 | **** |
| **IFNβ1** | 48 | 2019.Oax.10 vs. 2019.Oax.17 | 2.483 | 0.0685 | ns |
| **IFNβ1** | 48 | 2019.Oax.10 vs. 2019.Oax.19 | 3.12 | 0.4151 | ns |
| **IFNβ1** | 48 | 2019.Oax.10 vs. 2019.Oax.31 | 0.05377 | >0.9999 | ns |

| | | | | | |
|---|---|---|---|---|---|
| **IFNβ1** | 48 | 2019.Oax.10 vs. 2016.Oax.02 | 2.053 | 0.1810 | ns |
| **IFNβ1** | 48 | 2019.Oax.17 vs. 2019.Oax.19 | 0.6367 | 0.9875 | ns |
| **IFNβ1** | 48 | 2019.Oax.17 vs. 2019.Oax.31 | -2.43 | 0.4317 | ns |
| **IFNβ1** | 48 | 2019.Oax.17 vs. 2016.Oax.02 | -0.43 | 0.9564 | ns |

**Supplementary Table S3. (Continued)**

| Gene | Time (hpi) | Comparison | Mean difference | Adjusted p-value | Significance |
|---|---|---|---|---|---|
| **IFNβ1** | 48 | 2019.Oax.19 vs. 2019.Oax.31 | -3.066 | 0.5165 | ns |
| **IFNβ1** | 48 | 2019.Oax.19 vs. 2016.Oax.02 | -1.067 | 0.9392 | ns |
| **IFNβ1** | 48 | 2019.Oax.31 vs. 2016.Oax.02 | 2 | 0.5751 | ns |
| **IL8** | 6 | 2019.Oax.10 vs. 2019.Oax.17 | -3.457 | 0.0002 | *** |
| **IL8** | 6 | 2019.Oax.10 vs. 2019.Oax.19 | -0.5833 | 0.0657 | ns |
| **IL8** | 6 | 2019.Oax.10 vs. 2019.Oax.31 | 0.1967 | 0.9057 | ns |
| **IL8** | 6 | 2019.Oax.10 vs. 2016.Oax.02 | 1.483 | 0.2827 | ns |
| **IL8** | 6 | 2019.Oax.17 vs. 2019.Oax.19 | 2.873 | 0.0003 | *** |
| **IL8** | 6 | 2019.Oax.17 vs. 2019.Oax.31 | 3.653 | 0.0022 | ** |
| **IL8** | 6 | 2019.Oax.17 vs. 2016.Oax.02 | 4.94 | 0.0257 | * |
| **IL8** | 6 | 2019.Oax.19 vs. 2019.Oax.31 | 0.78 | 0.1808 | ns |
| **IL8** | 6 | 2019.Oax.19 vs. 2016.Oax.02 | 2.067 | 0.1575 | ns |
| **IL8** | 6 | 2019.Oax.31 vs. 2016.Oax.02 | 1.287 | 0.3540 | ns |
| **IL8** | 12 | 2019.Oax.10 vs. 2019.Oax.17 | 5.197 | 0.0008 | *** |
| **IL8** | 12 | 2019.Oax.10 vs. 2019.Oax.19 | 9.368 | 0.0003 | *** |
| **IL8** | 12 | 2019.Oax.10 vs. 2019.Oax.31 | 11.32 | <0.0001 | **** |
| **IL8** | 12 | 2019.Oax.10 vs. 2016.Oax.02 | 10.61 | 0.0089 | ** |
| **IL8** | 12 | 2019.Oax.17 vs. 2019.Oax.19 | 4.171 | 0.0099 | ** |
| **IL8** | 12 | 2019.Oax.17 vs. 2019.Oax.31 | 6.118 | <0.0001 | **** |
| **IL8** | 12 | 2019.Oax.17 vs. 2016.Oax.02 | 5.409 | 0.0462 | * |
| **IL8** | 12 | 2019.Oax.19 vs. 2019.Oax.31 | 1.947 | 0.0625 | ns |
| **IL8** | 12 | 2019.Oax.19 vs. 2016.Oax.02 | 1.238 | 0.5718 | ns |
| **IL8** | 12 | 2019.Oax.31 vs. 2016.Oax.02 | -0.7088 | 0.8351 | ns |
| **IL8** | 24 | 2019.Oax.10 vs. 2019.Oax.17 | 1.373 | 0.0603 | ns |
| **IL8** | 24 | 2019.Oax.10 vs. 2019.Oax.19 | -2.68 | 0.0117 | * |
| **IL8** | 24 | 2019.Oax.10 vs. 2019.Oax.31 | -13.15 | <0.0001 | **** |
| **IL8** | 24 | 2019.Oax.10 vs. 2016.Oax.02 | -8.381 | <0.0001 | **** |
| **IL8** | 24 | 2019.Oax.17 vs. 2019.Oax.19 | -4.053 | 0.0024 | ** |
| **IL8** | 24 | 2019.Oax.17 vs. 2019.Oax.31 | -14.53 | <0.0001 | **** |
| **IL8** | 24 | 2019.Oax.17 vs. 2016.Oax.02 | -9.754 | 0.0001 | *** |

| | | | | | |
|---|---|---|---|---|---|
| **IL8** | 24 | 2019.Oax.19 vs. 2019.Oax.31 | -10.47 | 0.0008 | *** |
| **IL8** | 24 | 2019.Oax.19 vs. 2016.Oax.02 | -5.701 | 0.0030 | ** |
| **IL8** | 24 | 2019.Oax.31 vs. 2016.Oax.02 | 4.772 | 0.0001 | *** |
| **IL8** | 48 | 2019.Oax.10 vs. 2019.Oax.17 | 2.567 | 0.0134 | * |

## Supplementary Table S3. (Continued)

| Gene | Time (hpi) | Comparison | Mean difference | Adjusted p-value | Significance |
|---|---|---|---|---|---|
| **IL8** | 48 | 2019.Oax.10 vs. 2019.Oax.19 | 1.977 | 0.5484 | ns |
| **IL8** | 48 | 2019.Oax.10 vs. 2019.Oax.31 | -2.97 | 0.0043 | ** |
| **IL8** | 48 | 2019.Oax.10 vs. 2016.Oax.02 | -4.457 | <0.0001 | **** |
| **IL8** | 48 | 2019.Oax.17 vs. 2019.Oax.19 | -0.59 | 0.9778 | ns |
| **IL8** | 48 | 2019.Oax.17 vs. 2019.Oax.31 | -5.536 | 0.0004 | *** |
| **IL8** | 48 | 2019.Oax.17 vs. 2016.Oax.02 | -7.023 | 0.0018 | ** |
| **IL8** | 48 | 2019.Oax.19 vs. 2019.Oax.31 | -4.946 | 0.1329 | ns |
| **IL8** | 48 | 2019.Oax.19 vs. 2016.Oax.02 | -6.433 | 0.0857 | ns |
| **IL8** | 48 | 2019.Oax.31 vs. 2016.Oax.02 | -1.487 | 0.0402 | * |
| **TNFα** | 6 | 2019.Oax.10 vs. 2019.Oax.17 | 10.24 | <0.0001 | **** |
| **TNFα** | 6 | 2019.Oax.10 vs. 2019.Oax.19 | 13.63 | 0.0001 | *** |
| **TNFα** | 6 | 2019.Oax.10 vs. 2019.Oax.31 | 12.81 | <0.0001 | **** |
| **TNFα** | 6 | 2019.Oax.10 vs. 2016.Oax.02 | 6.833 | 0.0130 | * |
| **TNFα** | 6 | 2019.Oax.17 vs. 2019.Oax.19 | 3.39 | 0.0097 | ** |
| **TNFα** | 6 | 2019.Oax.17 vs. 2019.Oax.31 | 2.567 | 0.0147 | * |
| **TNFα** | 6 | 2019.Oax.17 vs. 2016.Oax.02 | -3.41 | 0.0351 | * |
| **TNFα** | 6 | 2019.Oax.19 vs. 2019.Oax.31 | -0.8233 | 0.4241 | ns |
| **TNFα** | 6 | 2019.Oax.19 vs. 2016.Oax.02 | -6.8 | 0.0020 | ** |
| **TNFα** | 6 | 2019.Oax.31 vs. 2016.Oax.02 | -5.977 | 0.0037 | ** |
| **TNFα** | 12 | 2019.Oax.10 vs. 2019.Oax.17 | -17.08 | <0.0001 | **** |
| **TNFα** | 12 | 2019.Oax.10 vs. 2019.Oax.19 | -14.79 | <0.0001 | **** |
| **TNFα** | 12 | 2019.Oax.10 vs. 2019.Oax.31 | -2.238 | 0.0096 | ** |
| **TNFα** | 12 | 2019.Oax.10 vs. 2016.Oax.02 | -1.014 | 0.2010 | ns |
| **TNFα** | 12 | 2019.Oax.17 vs. 2019.Oax.19 | 2.291 | 0.0005 | *** |
| **TNFα** | 12 | 2019.Oax.17 vs. 2019.Oax.31 | 14.84 | <0.0001 | **** |
| **TNFα** | 12 | 2019.Oax.17 vs. 2016.Oax.02 | 16.07 | <0.0001 | **** |
| **TNFα** | 12 | 2019.Oax.19 vs. 2019.Oax.31 | 12.55 | <0.0001 | **** |
| **TNFα** | 12 | 2019.Oax.19 vs. 2016.Oax.02 | 13.78 | <0.0001 | **** |
| **TNFα** | 12 | 2019.Oax.31 vs. 2016.Oax.02 | 1.225 | 0.1200 | ns |
| **TNFα** | 24 | 2019.Oax.10 vs. 2019.Oax.17 | 2.097 | 0.0245 | * |

| | | | | | |
|---|---|---|---|---|---|
| **TNFα** | 24 | 2019.Oax.10 vs. 2019.Oax.19 | -0.1233 | 0.9412 | ns |
| **TNFα** | 24 | 2019.Oax.10 vs. 2019.Oax.31 | -4.13 | 0.0003 | *** |
| **TNFα** | 24 | 2019.Oax.10 vs. 2016.Oax.02 | -10.87 | <0.0001 | **** |
| **TNFα** | 24 | 2019.Oax.17 vs. 2019.Oax.19 | -2.22 | 0.0053 | ** |

## Supplementary Table S3. (Continued)

| Gene | Time (hpi) | Comparison | Mean difference | Adjusted p-value | Significance |
|---|---|---|---|---|---|
| **TNFα** | 24 | 2019.Oax.17 vs. 2019.Oax.31 | -6.226 | 0.0004 | *** |
| **TNFα** | 24 | 2019.Oax.17 vs. 2016.Oax.02 | -12.96 | <0.0001 | **** |
| **TNFα** | 24 | 2019.Oax.19 vs. 2019.Oax.31 | -4.006 | 0.0008 | *** |
| **TNFα** | 24 | 2019.Oax.19 vs. 2016.Oax.02 | -10.74 | <0.0001 | **** |
| **TNFα** | 24 | 2019.Oax.31 vs. 2016.Oax.02 | -6.738 | 0.0001 | *** |
| **TNFα** | 48 | 2019.Oax.10 vs. 2019.Oax.17 | 6.013 | 0.0007 | *** |
| **TNFα** | 48 | 2019.Oax.10 vs. 2019.Oax.19 | -3.567 | 0.0060 | ** |
| **TNFα** | 48 | 2019.Oax.10 vs. 2019.Oax.31 | -10.11 | 0.0003 | *** |
| **TNFα** | 48 | 2019.Oax.10 vs. 2016.Oax.02 | -12.96 | 0.0002 | *** |
| **TNFα** | 48 | 2019.Oax.17 vs. 2019.Oax.19 | -9.58 | 0.0011 | ** |
| **TNFα** | 48 | 2019.Oax.17 vs. 2019.Oax.31 | -16.12 | <0.0001 | **** |
| **TNFα** | 48 | 2019.Oax.17 vs. 2016.Oax.02 | -18.97 | <0.0001 | **** |
| **TNFα** | 48 | 2019.Oax.19 vs. 2019.Oax.31 | -6.543 | <0.0001 | **** |
| **TNFα** | 48 | 2019.Oax.19 vs. 2016.Oax.02 | -9.393 | 0.0033 | ** |
| **TNFα** | 48 | 2019.Oax.31 vs. 2016.Oax.02 | -2.85 | 0.0546 | ns |
| **RANTES** | 6 | 2019.Oax.10 vs. 2019.Oax.17 | 4.017 | <0.0001 | **** |
| **RANTES** | 6 | 2019.Oax.10 vs. 2019.Oax.19 | 9.727 | <0.0001 | **** |
| **RANTES** | 6 | 2019.Oax.10 vs. 2019.Oax.31 | 10.24 | <0.0001 | **** |
| **RANTES** | 6 | 2019.Oax.10 vs. 2016.Oax.02 | 7.253 | 0.0051 | ** |
| **RANTES** | 6 | 2019.Oax.17 vs. 2019.Oax.19 | 5.71 | <0.0001 | **** |
| **RANTES** | 6 | 2019.Oax.17 vs. 2019.Oax.31 | 6.223 | <0.0001 | **** |
| **RANTES** | 6 | 2019.Oax.17 vs. 2016.Oax.02 | 3.237 | 0.0160 | * |
| **RANTES** | 6 | 2019.Oax.19 vs. 2019.Oax.31 | 0.5133 | 0.0164 | * |
| **RANTES** | 6 | 2019.Oax.19 vs. 2016.Oax.02 | -2.473 | 0.0137 | * |
| **RANTES** | 6 | 2019.Oax.31 vs. 2016.Oax.02 | -2.987 | 0.0058 | ** |
| **RANTES** | 12 | 2019.Oax.10 vs. 2019.Oax.17 | -7.389 | <0.0001 | **** |
| **RANTES** | 12 | 2019.Oax.10 vs. 2019.Oax.19 | 12.36 | 0.0003 | *** |
| **RANTES** | 12 | 2019.Oax.10 vs. 2019.Oax.31 | 13.05 | <0.0001 | **** |
| **RANTES** | 12 | 2019.Oax.10 vs. 2016.Oax.02 | 10.67 | <0.0001 | **** |
| **RANTES** | 12 | 2019.Oax.17 vs. 2019.Oax.19 | 19.75 | <0.0001 | **** |

| | | | | | |
|---|---|---|---|---|---|
| **RANTES** | 12 | 2019.Oax.17 vs. 2019.Oax.31 | 20.44 | <0.0001 | **** |
| **RANTES** | 12 | 2019.Oax.17 vs. 2016.Oax.02 | 18.06 | <0.0001 | **** |
| **RANTES** | 12 | 2019.Oax.19 vs. 2019.Oax.31 | 0.6905 | 0.3683 | ns |
| **RANTES** | 12 | 2019.Oax.19 vs. 2016.Oax.02 | -1.692 | 0.1894 | ns |

## Supplementary Table S3. (Continued)

| Gene | Time (hpi) | Comparison | Mean difference | Adjusted p-value | Significance |
|---|---|---|---|---|---|
| **RANTES** | 12 | 2019.Oax.31 vs. 2016.Oax.02 | -2.382 | 0.0157 | * |
| **RANTES** | 24 | 2019.Oax.10 vs. 2019.Oax.17 | 2.07 | 0.1589 | ns |
| **RANTES** | 24 | 2019.Oax.10 vs. 2019.Oax.19 | 2.19 | 0.0384 | * |
| **RANTES** | 24 | 2019.Oax.10 vs. 2019.Oax.31 | -1.28 | <0.0001 | **** |
| **RANTES** | 24 | 2019.Oax.10 vs. 2016.Oax.02 | -0.8009 | 0.4259 | ns |
| **RANTES** | 24 | 2019.Oax.17 vs. 2019.Oax.19 | 0.12 | 0.9831 | ns |
| **RANTES** | 24 | 2019.Oax.17 vs. 2019.Oax.31 | -3.35 | 0.0618 | ns |
| **RANTES** | 24 | 2019.Oax.17 vs. 2016.Oax.02 | -2.871 | 0.0074 | ** |
| **RANTES** | 24 | 2019.Oax.19 vs. 2019.Oax.31 | -3.47 | 0.0139 | * |
| **RANTES** | 24 | 2019.Oax.19 vs. 2016.Oax.02 | -2.991 | 0.0047 | ** |
| **RANTES** | 24 | 2019.Oax.31 vs. 2016.Oax.02 | 0.4786 | 0.6958 | ns |
| **RANTES** | 48 | 2019.Oax.10 vs. 2019.Oax.17 | -0.5967 | 0.5681 | ns |
| **RANTES** | 48 | 2019.Oax.10 vs. 2019.Oax.19 | 0.2767 | 0.5774 | ns |
| **RANTES** | 48 | 2019.Oax.10 vs. 2019.Oax.31 | -15.92 | <0.0001 | **** |
| **RANTES** | 48 | 2019.Oax.10 vs. 2016.Oax.02 | -1.123 | 0.0219 | * |
| **RANTES** | 48 | 2019.Oax.17 vs. 2019.Oax.19 | 0.8733 | 0.1245 | ns |
| **RANTES** | 48 | 2019.Oax.17 vs. 2019.Oax.31 | -15.32 | <0.0001 | **** |
| **RANTES** | 48 | 2019.Oax.17 vs. 2016.Oax.02 | -0.5266 | 0.7687 | ns |
| **RANTES** | 48 | 2019.Oax.19 vs. 2019.Oax.31 | -16.2 | <0.0001 | **** |
| **RANTES** | 48 | 2019.Oax.19 vs. 2016.Oax.02 | -1.4 | 0.0982 | ns |
| **RANTES** | 48 | 2019.Oax.31 vs. 2016.Oax.02 | 14.8 | <0.0001 | **** |
| **MCP-1** | 6 | 2019.Oax.10 vs. 2019.Oax.17 | 3.033 | 0.0027 | ** |
| **MCP-1** | 6 | 2019.Oax.10 vs. 2019.Oax.19 | 6.95 | <0.0001 | **** |
| **MCP-1** | 6 | 2019.Oax.10 vs. 2019.Oax.31 | 7.633 | <0.0001 | **** |
| **MCP-1** | 6 | 2019.Oax.10 vs. 2016.Oax.02 | 5.62 | 0.0016 | ** |
| **MCP-1** | 6 | 2019.Oax.17 vs. 2019.Oax.19 | 3.917 | 0.0010 | ** |
| **MCP-1** | 6 | 2019.Oax.17 vs. 2019.Oax.31 | 4.6 | 0.0073 | ** |
| **MCP-1** | 6 | 2019.Oax.17 vs. 2016.Oax.02 | 2.587 | 0.0110 | * |
| **MCP-1** | 6 | 2019.Oax.19 vs. 2019.Oax.31 | 0.683 | 0.1424 | ns |
| **MCP-1** | 6 | 2019.Oax.19 vs. 2016.Oax.02 | -1.33 | 0.0917 | ns |

| | | | | | |
|---|---|---|---|---|---|
| **MCP-1** | 6 | 2019.Oax.31 vs. 2016.Oax.02 | -2.013 | 0.0580 | ns |
| **MCP-1** | 12 | 2019.Oax.10 vs. 2019.Oax.17 | -3.109 | 0.0071 | ** |
| **MCP-1** | 12 | 2019.Oax.10 vs. 2019.Oax.19 | -0.986 | 0.5866 | ns |
| **MCP-1** | 12 | 2019.Oax.10 vs. 2019.Oax.31 | 0.045 | 0.9992 | ns |

**Supplementary Table S3. (Continued)**

| Gene | Time (hpi) | Comparison | Mean difference | Adjusted p-value | Significance |
|---|---|---|---|---|---|
| **MCP-1** | 12 | 2019.Oax.10 vs. 2016.Oax.02 | -0.601 | 0.8609 | ns |
| **MCP-1** | 12 | 2019.Oax.17 vs. 2019.Oax.19 | 2.124 | 0.1875 | ns |
| **MCP-1** | 12 | 2019.Oax.17 vs. 2019.Oax.31 | 3.154 | 0.0004 | *** |
| **MCP-1** | 12 | 2019.Oax.17 vs. 2016.Oax.02 | 2.509 | 0.1483 | ns |
| **MCP-1** | 12 | 2019.Oax.19 vs. 2019.Oax.31 | 1.03 | 0.5385 | ns |
| **MCP-1** | 12 | 2019.Oax.19 vs. 2016.Oax.02 | 0.385 | 0.9868 | ns |
| **MCP-1** | 12 | 2019.Oax.31 vs. 2016.Oax.02 | -0.646 | 0.8073 | ns |
| **MCP-1** | 24 | 2019.Oax.10 vs. 2019.Oax.17 | 1.093 | 0.0007 | *** |
| **MCP-1** | 24 | 2019.Oax.10 vs. 2019.Oax.19 | 0.59 | 0.0655 | ns |
| **MCP-1** | 24 | 2019.Oax.10 vs. 2019.Oax.31 | 4.43 | 0.0017 | ** |
| **MCP-1** | 24 | 2019.Oax.10 vs. 2016.Oax.02 | 4.679 | <0.0001 | **** |
| **MCP-1** | 24 | 2019.Oax.17 vs. 2019.Oax.19 | -0.503 | 0.1029 | ns |
| **MCP-1** | 24 | 2019.Oax.17 vs. 2019.Oax.31 | 3.337 | 0.0043 | ** |
| **MCP-1** | 24 | 2019.Oax.17 vs. 2016.Oax.02 | 3.586 | <0.0001 | **** |
| **MCP-1** | 24 | 2019.Oax.19 vs. 2019.Oax.31 | 3.84 | 0.0005 | *** |
| **MCP-1** | 24 | 2019.Oax.19 vs. 2016.Oax.02 | 4.089 | 0.0002 | *** |
| **MCP-1** | 24 | 2019.Oax.31 vs. 2016.Oax.02 | 0.249 | 0.6546 | ns |
| **MCP-1** | 48 | 2019.Oax.10 vs. 2019.Oax.17 | -0.327 | 0.8359 | ns |
| **MCP-1** | 48 | 2019.Oax.10 vs. 2019.Oax.19 | 0.03 | >0.9999 | ns |
| **MCP-1** | 48 | 2019.Oax.10 vs. 2019.Oax.31 | 1.27 | 0.4648 | ns |
| **MCP-1** | 48 | 2019.Oax.10 vs. 2016.Oax.02 | 0.56 | 0.5618 | ns |
| **MCP-1** | 48 | 2019.Oax.17 vs. 2019.Oax.19 | 0.357 | 0.9982 | ns |
| **MCP-1** | 48 | 2019.Oax.17 vs. 2019.Oax.31 | 1.597 | 0.3463 | ns |
| **MCP-1** | 48 | 2019.Oax.17 vs. 2016.Oax.02 | 0.887 | 0.4194 | ns |
| **MCP-1** | 48 | 2019.Oax.19 vs. 2019.Oax.31 | 1.24 | 0.8997 | ns |
| **MCP-1** | 48 | 2019.Oax.19 vs. 2016.Oax.02 | 0.53 | 0.9919 | ns |
| **MCP-1** | 48 | 2019.Oax.31 vs. 2016.Oax.02 | -0.71 | 0.8378 | ns |

Notes: Relative gene-expression data were analyzed using two-way analysis of variance (ANOVA), followed by Tukey's multiple-comparisons test. Comparisons were performed only among the viral isolates at each time point. IFN-α A/D and LPS were included as positive controls for antiviral and inflammatory responses, respectively, but were not included in the

multiple-comparisons analysis. Mean differences, adjusted p-values, and significance levels are reported. Statistical significance was defined as follows: ns, not significant ($p \geq 0.05$); * $p < 0.05$; ** $p < 0.01$; *** $p < 0.001$; and **** $p < 0.0001$.

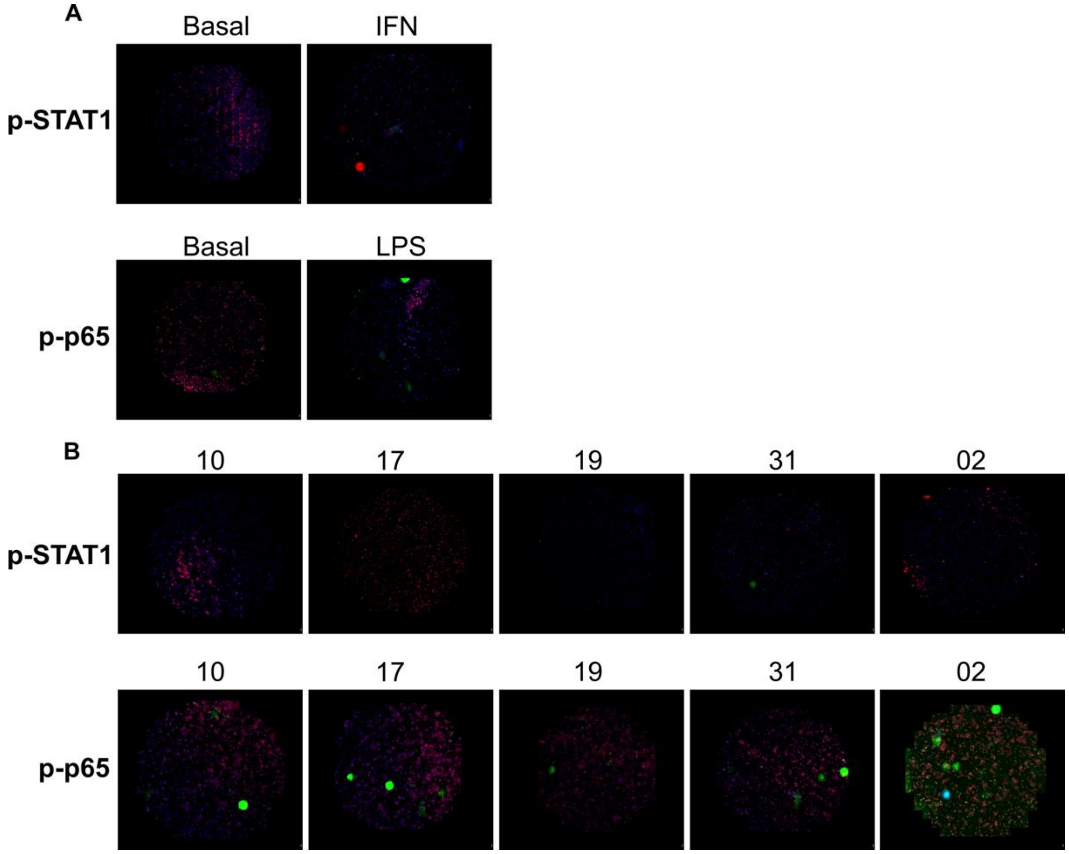


**Supplementary Figure S2.** Representative whole-slide fluorescence images used for automated quantitative immunofluorescence analysis.
Representative whole-slide fluorescence scans acquired using a Zeiss AxioScan Z.1 digital slide scanner. These images illustrate the complete acquisition area analyzed for quantitative immunofluorescence. Image preprocessing included illumination correction (BaSiC), image registration (ASHLAR), and single-cell segmentation using StarDist. Quantitative fluorescence measurements presented in Figure 5 were obtained from all segmented cells within each whole-slide image rather than manually selected microscopic fields.

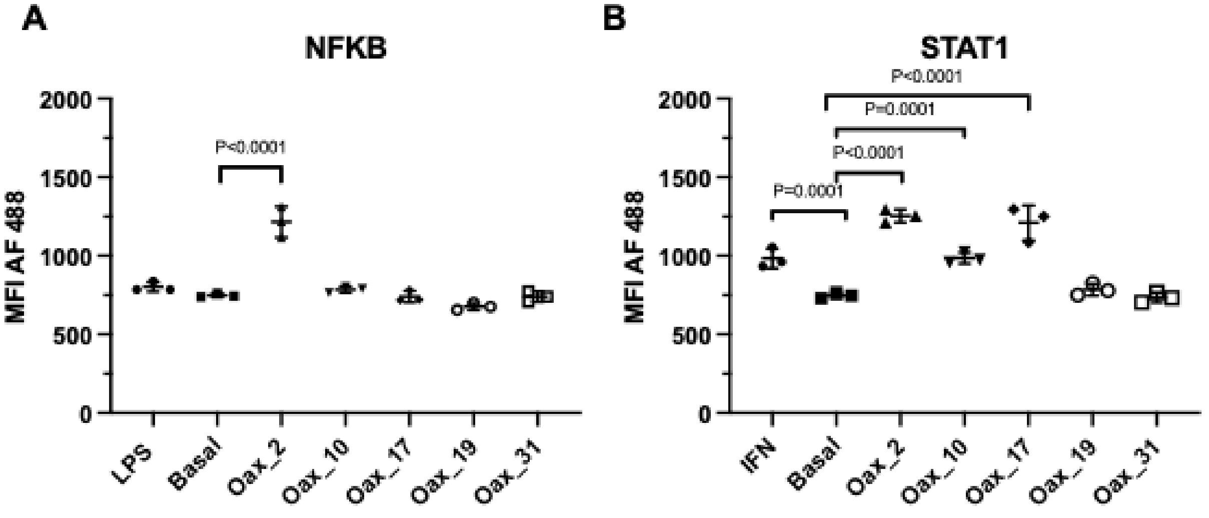


**Supplementary Figure S3.** Biological replicate-level median fluorescence intensity values for phosphorylated STAT1 (A) and phosphorylated NF-κB p65 (B). Each symbol represents the median fluorescence intensity obtained from one independent biological replicate. Statistical comparisons were performed using one-way ANOVA followed by Fisher's uncorrected LSD multiple-comparison test. Exact unadjusted p-values are summarized in Supplementary Table S4.

**Supplementary Table S4.** Pairwise statistical comparisons of phosphorylated STAT1 median fluorescence intensity among experimental groups. Statistical comparisons were performed using one-way ANOVA followed by Fisher's uncorrected least significant difference (LSD) multiple-comparison test. Biological replicates (n = 3 per group) were used as the experimental unit. The table reports the mean difference, 95% confidence interval (CI), and exact unadjusted p-value for each pairwise comparison. Corresponding biological replicate-level median fluorescence intensity values are shown in Supplementary Figure S3A.

| p-STAT1 | Mean difference. | 95% CI | Summary | Exact P value |
|---|---|---|---|---|
| **Basal vs. IFN** | -235.6 | -333.5 to -137.7 | *** | 0.0001 |
| **Basal vs. Oax_2** | -506.8 | -604.6 to -408.9 | **** | <0.0001 |
| **Basal vs. Oax_10** | -237.7 | -335.6 to -139.9 | *** | 0.0001 |
| **Basal vs. Oax_17** | -462.4 | -560.3 to -364.6 | **** | <0.0001 |
| **Basal vs. Oax_19** | -37.27 | -135.2 to 60.62 | ns | 0.4279 |
| **Basal vs. Oax_31** | 13.36 | -84.53 to 111.3 | ns | 0.7740 |

**Supplementary Table S5.** Pairwise statistical comparisons of phosphorylated NF-κB p65 median fluorescence intensity among experimental groups. Statistical comparisons were performed using one-way ANOVA followed by Fisher's uncorrected least significant difference (LSD) multiple-comparison test. Biological replicates (n = 3 per group) were used as the experimental unit. The table reports the mean difference, 95% confidence interval (CI), and exact unadjusted p-value for

each pairwise comparison. Corresponding biological replicate-level median fluorescence intensity values are shown in Supplementary Figure S3B.

| NFκB p-65 | Mean difference. | 95% CI | Summary | Exact P value |
|---|---|---|---|---|
| **Basal vs. LPS** | -55.01 | -133.5 to 23.44 | ns | 0.1548 |
| **Basal vs. Oax_2** | -467.2 | -545.7 to -388.8 | **** | <0.0001 |
| **Basal vs. Oax_10** | -37.42 | -115.9 to 41.03 | ns | 0.3236 |
| **Basal vs. Oax_17** | 8.389 | -70.06 to 86.84 | ns | 0.8219 |
| **Basal vs. Oax_19** | 70.83 | -7.624 to 149.3 | ns | 0.0733 |
| **Basal vs. Oax_31** | 8.001 | -70.45 to 86.45 | ns | 0.8300 |
